# Factors Influencing Adherence to mHealth Apps for Prevention or Management of Noncommunicable Diseases: Systematic Review

**DOI:** 10.2196/35371

**Published:** 2022-05-25

**Authors:** Robert Jakob, Samira Harperink, Aaron Maria Rudolf, Elgar Fleisch, Severin Haug, Jacqueline Louise Mair, Alicia Salamanca-Sanabria, Tobias Kowatsch

**Affiliations:** 1 Centre for Digital Health Interventions Department of Management, Technology and Economics ETH Zurich Zurich Switzerland; 2 Centre for Digital Health Interventions Institute of Technology Management University of St. Gallen St. Gallen Switzerland; 3 Future Health Technologies, Singapore-ETH Centre Campus for Research Excellence And Technological Enterprise Singapore Singapore; 4 Swiss Research Institute for Public Health and Addiction Zurich University Zurich Switzerland; 5 Saw Swee Hock School of Public Health National University of Singapore Singapore Singapore

**Keywords:** intended use, adherence, engagement, attrition, retention, mHealth, eHealth, digital health intervention, noncommunicable disease, NCD, mobile phone

## Abstract

**Background:**

Mobile health (mHealth) apps show vast potential in supporting patients and health care systems with the increasing prevalence and economic costs of noncommunicable diseases (NCDs) worldwide. However, despite the availability of evidence-based mHealth apps, a substantial proportion of users do not adhere to them as intended and may consequently not receive treatment. Therefore, understanding the factors that act as barriers to or facilitators of adherence is a fundamental concern in preventing intervention dropouts and increasing the effectiveness of digital health interventions.

**Objective:**

This review aimed to help stakeholders develop more effective digital health interventions by identifying factors influencing the continued use of mHealth apps targeting NCDs. We further derived quantified adherence scores for various health domains to validate the qualitative findings and explore adherence benchmarks.

**Methods:**

A comprehensive systematic literature search (January 2007 to December 2020) was conducted on MEDLINE, Embase, Web of Science, Scopus, and ACM Digital Library. Data on intended use, actual use, and factors influencing adherence were extracted. Intervention-related and patient-related factors with a positive or negative influence on adherence are presented separately for the health domains of NCD self-management, mental health, substance use, nutrition, physical activity, weight loss, multicomponent lifestyle interventions, mindfulness, and other NCDs. Quantified adherence measures, calculated as the ratio between the estimated intended use and actual use, were derived for each study and compared with the qualitative findings.

**Results:**

The literature search yielded 2862 potentially relevant articles, of which 99 (3.46%) were included as part of the inclusion criteria. A total of 4 intervention-related factors indicated positive effects on adherence across all health domains: personalization or tailoring of the content of mHealth apps to the individual needs of the user, reminders in the form of individualized push notifications, user-friendly and technically stable app design, and personal support complementary to the digital intervention. Social and gamification features were also identified as drivers of app adherence across several health domains. A wide variety of patient-related factors such as user characteristics or recruitment channels further affects adherence. The derived adherence scores of the included mHealth apps averaged 56.0% (SD 24.4%).

**Conclusions:**

This study contributes to the scarce scientific evidence on factors that positively or negatively influence adherence to mHealth apps and is the first to quantitatively compare adherence relative to the intended use of various health domains. As underlying studies mostly have a pilot character with short study durations, research on factors influencing adherence to mHealth apps is still limited. To facilitate future research on mHealth app adherence, researchers should clearly outline and justify the app’s intended use; report objective data on actual use relative to the intended use; and, ideally, provide long-term use and retention data.

## Introduction

### Rationale

Digital health interventions (DHIs) show vast potential in supporting patients and health care systems with the globally increasing prevalence and economic costs of noncommunicable diseases (NCDs), which is the leading causes of death and disability worldwide [1,2]. More specifically, mobile health (mHealth) apps are now considered accessible and scalable solutions to promoting behavior change among patients, improving health outcomes, and reducing health care costs [3-5]. Correspondingly, the number of available mHealth apps has continuously grown to >300.000, with approximately 200 new mHealth apps released each day [2,6].

However, despite increasing evidence and availability, mHealth apps are subject to significant dropout rates, with a substantial proportion of users not adhering to them as intended [7,8]. Recent research has shown that up to 80% of all participants in mHealth interventions only engage at a minimum level, do not log into the mHealth app more than once, and do not consistently use the app in the long term [9]. Another study examining mHealth app use in more extensive real-world settings reported low retention rates, with only 3.9% of participants using mHealth apps for >15 days [10]. The reported low adherence and high attrition levels further highlight the necessity of developing more effective models, best practices, and interventions [8,11].

As nonadherence relative to intended use jeopardizes treatment success and, thus, might lead to an increased number of hospitalizations, it is considered a fundamental concern in the development of mHealth apps [8,12-15]. However, the scientific body of literature lacks concise conceptualizations and measures for the intended use of mHealth apps, whereas intervention components and factors influencing adherence remain to be explored [13,16]. Following previous studies, we define *adherence* as “the degree to which the user followed the program as it was designed,” which can be paraphrased as “adherence relative to the intended use” [13,17,18].

With smartphone apps being the primary intervention component, adherence relative to the intended use is principally informed by user acceptance and the use of information technology [19]. Previous research underscores the necessity of mHealth apps that must be first accepted and used in an intended way to then achieve a desired health behavior change [3,19]. Correspondingly, previous research has identified factors affecting the uptake of and engagement with health and well-being smartphone apps [11,20-23]. Many of these strategies and factors, such as well-designed reminders, self-monitoring features, and embedded health professional support, have been applied across various health domains [11,20,21]. Some of these factors, such as reminders, can be further applied as retention methods and strategies for cohort studies in general and may thus extend the scope of DHIs [24,25].

Identifying the factors that influence adherence relative to intended use may support and extend these findings. Given previous research and their relation to technology use and acceptance, we can assume that these factors may be not only generalizable across various health behavior domains but also be applicable to DHIs using alternative information technologies. To our knowledge, no systematic review has been conducted on the factors influencing adherence to mHealth apps designed to prevent or manage NCDs. Furthermore, to the best of our knowledge, no review has previously explored the quantifying of adherence to assess qualitatively identified factors.

### Objectives

Preventing intervention dropouts and thus increasing the effectiveness of mHealth apps requires an understanding of the factors that act as barriers to or facilitators of intervention adherence. This review aimed to identify factors influencing adherence relative to the intended use of mHealth apps, which may help stakeholders better plan, develop, and evaluate mHealth apps. To help readers navigate through the identified factors, we further categorized them into intervention-related factors that app developers can potentially improve upon through product changes (eg, the inclusion of certain app features) and patient-related factors that are hardly adjustable (eg, user characteristics). These factors were separated into their potential positive or negative influences on adherence.

In the absence of a universally agreed-upon approach to measuring adherence to mHealth interventions, we exploratively derived an adherence score as the ratio between the intended and actual use of each study to describe adherence quantitatively and consistently. The primary aim of the resulting adherence score was to quantitatively assess the findings from the qualitative extraction of factors influencing adherence. As the intended use varies substantially across different mHealth apps, we extracted the intended use for each included mHealth app individually. We then compared the intended use with the actual use reported in the corresponding study. To the best of our knowledge, this exploratory approach of a quantified adherence score has not been applied previously.

In summary, this review aimed to answer the following research questions:

1. Which intervention-related factors influence adherence relative to the intended use of mHealth apps targeting NCDs in adults?
2. Which patient-related factors influence adherence relative to the intended use of mHealth apps targeting NCDs in adults?
3. How do the adherence rates of mHealth apps for NCDs compare across different health domains?

## Methods

### Database Selection and Search Strategy

This review was conducted according to the PRISMA (Preferred Reporting Items for Systematic Reviews and Meta-Analyses; Multimedia Appendix 1). A review protocol was submitted to the Federal Office of Public Health of the Swiss Confederation on October 7, 2020, but was not publicly registered.

The electronic databases Embase (including MEDLINE and PubMed), Web of Science, Scopus, and ACM Digital Library were searched using a predefined search strategy that included search terms related to mHealth apps, app use, and study design (Multimedia Appendix 2). The search terms were customized for each electronic database, and if the respective database allowed it, the corresponding Medical Subject Heading terms or topics were also integrated. Articles published in English between June 2007 (release of the iPhone) and December 2020, which focused on adult populations, were included. Studies that focused on communicable diseases were excluded. The inclusion and exclusion criteria listed in Textbox 1 were used to identify relevant articles.

**List of eligibility criteria (Population, Intervention, Comparison, Outcomes, and Study component [PICOS] along with inclusion and exclusion criteria and applied filters).**

**Inclusion criteria**

- Participants: adults aged ≥18 years; studies that included individuals aged ≥16 years were included if at least 70% of the participants were aged ≥18 years
- Intervention and context: studies investigating digital interventions that aimed to change ≥1 health behavior and the stated goal of the intervention was to prevent or treat a noncommunicable disease or condition
- Comparison: any kind of comparison
- Outcomes
  - Qualitative: factors predicting adherence or nonadherence relative to the intended use
  - Quantitative: information on the actual and intended use of the intervention or information on adherence relative to the intended use
- Study design: primary and secondary studies, including randomized controlled trials, systematic reviews, meta-analyses, observational studies, single-center experiments, feasibility studies, pilot studies, and experimental studies

**Exclusion criteria**

- Participants: children and adolescents aged <18 years and animals
- Intervention and context:
  - Studies with the smartphone not being the primary intervention component
  - Interventions not targeting noncommunicable diseases; for example, communicable diseases (influenza, norovirus, Ebola, and COVID-19)
- Comparison: none
- Outcomes: the study does not contain information on the actual and intended use of the intervention
- Study design: animal and laboratory studies, case reports, case series, narrative reviews, expert opinions, editorials, conference abstracts, and study protocols

**Applied filters**

- Time: studies published from June 2007 onward
- Language: English
- Access: open access or via institutional log-in

### Screening Process and Eligibility Criteria

**Figure 1:**
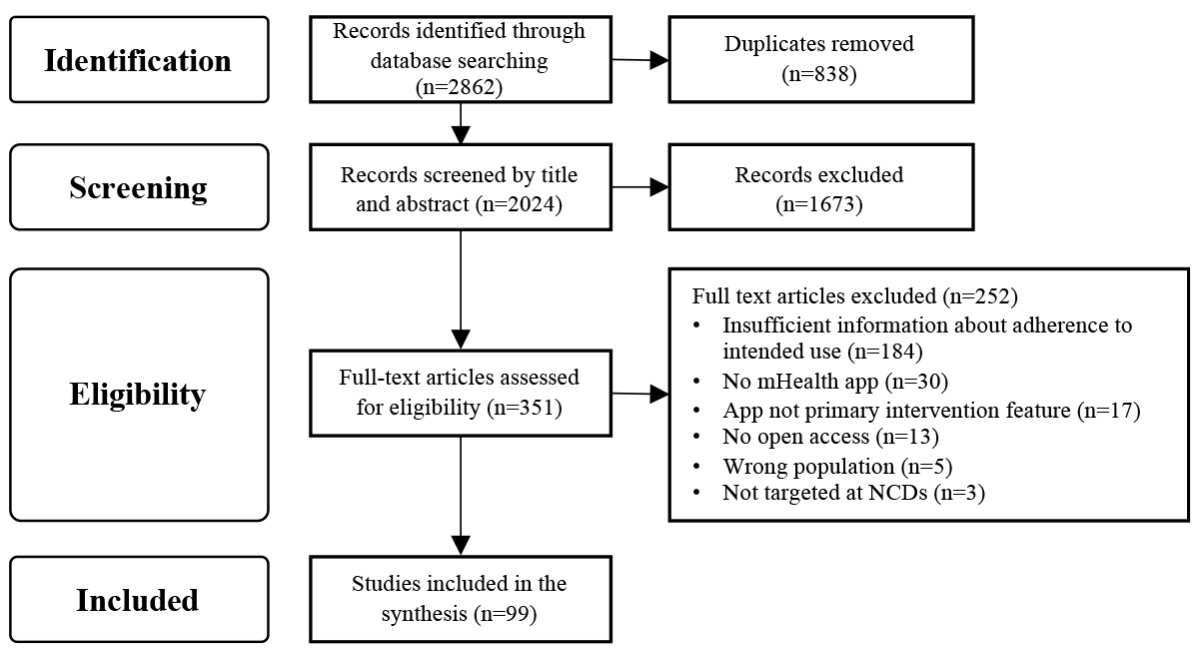
PRISMA (Preferred Reporting Items for Systematic Reviews and Meta-Analyses) flowchart illustrating the inclusion and exclusion of studies. mHealth: mobile health; NCD: noncommunicable disease.

The selection of publications was conducted in several steps (Figure 1). First, potentially relevant publications were identified by searching the literature databases. After excluding duplicates, titles and abstracts were independently reviewed by 3 researchers (SH, RJ, and AMR) according to the inclusion and exclusion criteria listed in Textbox 1. Disagreements were resolved through discussion. In a second screening step, the full texts of relevant articles were independently reviewed by 4 researchers (SH, RJ, AMR, and JLM) concerning the fulfillment of the inclusion and exclusion criteria. Discrepancies were again resolved through discussions. The web-based program Covidence (Veritas Health Innovation) was used for the entire process. After applying the search strategies outlined in Multimedia Appendix 2, the resulting database reference lists were imported into the Covidence database. The following Covidence features were used in the process: duplicate removal, title and abstract screening, full-text review, and export of PRISMA flowchart.

### Data Extraction

The following information was extracted for each included study: general study characteristics, study population characteristics, intervention characteristics, factors influencing adherence relative to intended use, and information on app use (Multimedia Appendix 3 [20,26-123]).

General study characteristics included the title, first author, year of publication, journal name, country, study design, and health domain.

Study population characteristics comprised age, gender, type of population (clinical or general population), type of disease, and number of study participants.

Intervention characteristics were app name, smartphone operating system (universal, Android only, or iOS only), type of mHealth app offered (publicly available or research apps), app developer (private company or nonprofit organization), level of personal support (no personal support during the intervention or continuous personal support), external monetary incentives and their value in US dollars, intervention duration in days, and effectiveness of the intervention in terms of health outcomes.

Factors influencing adherence relative to intended use were extracted and characterized as intervention-related factors (factors that developers can potentially improve through product changes) or patient-related factors (factors that can hardly be influenced by app developers, such as user characteristics). These factors were further categorized based on their positive or negative influence on adherence.

Information on app use comprised intended app use, actual app use, the number of intended intervention interactions, and interaction frequency (eg, daily or weekly intended use). The adherence score was defined as *adherence relative to the intended use* and was derived as the quantified ratio of intended use to actual use.

### Synthesis and Statistical Analyses

As a first step, the identified studies were categorized based on the mHealth app they investigated as follows: apps targeting NCD self-management (including the four main NCDs: asthma, diabetes, cardiovascular disease [CVD], and cancer), mental health disorders (anxiety and depression), substance use disorders (alcohol and tobacco), and behavioral risk factors (nutrition, physical activity, and weight loss). The categories were then further refined into the following health domains: NCD self-management (asthma management, diabetes management, CVD management, cancer management, and medication adherence), mental health (anxiety, depression, and multidisciplinary and others), substance use (alcohol, tobacco, and multidisciplinary and others), nutrition, physical activity, weight loss, multicomponent lifestyle interventions, and other NCDs.

In the second step, intervention-related and patient-related factors that were outlined in the studies as barriers to or facilitators of adherence were qualitatively evaluated, summarized within the health domain, and categorized based on their positive or negative influence.

In the third step, the adherence score was derived for each study focusing directly on a specific mHealth app (97/99, 98%) and was calculated as the ratio of intended use to actual use. The mean adherence scores were calculated for each health domain.

In the fourth step, correlations of adherence scores with other extracted variables were examined, and where possible, the qualitative results from step 2 were quantitatively compared for each health domain. Quantitative analyses were performed using SPSS Statistics (version 27; IBM Corp). Correlations of adherence scores with continuous variables (eg, the average age of study participants) were calculated using the Pearson correlation. Correlations with ordinal variables (eg, level of personal support) were calculated using the Spearman correlation.

Finally, a list of universally relevant factors with recommendations for the development and evaluation of mHealth apps was developed.

## Results

### Selection and Inclusion of Studies

The search of electronic databases was performed on January 3, 2021, and yielded 2862 articles. After excluding duplicates, 70.72% (2024/2862) of publications remained for the title and abstract screening. Subsequently, the full texts of 17.34% (351/2024) of articles were examined. Of the 351 studies, 99 (28.2%) were finally included in the data synthesis. Figure 1 visualizes the selection process and reasons for exclusion.

### Characteristics of Included Studies

In total, 99 studies were included in this review. Of these 99 studies, 2 (2%) were systematic reviews, and 97 (98%) evaluated specific mHealth apps. Randomized controlled trials were the most frequent study type (35/97, 36%), followed by pilot trials (31/97, 32%), pilot randomized controlled trials (12/97, 12%), cohort studies, mixed methods studies, and observational studies (each 5/97, 5%). Most of the studies were conducted in North America (37/97, 38%), followed by Europe (34/97, 35%), Australia (15/97, 15%), and Asia (10/97, 10%). Of the 97 studies, 90 (93%) were published within the past 5 years: 46 (47%) in 2020, 12 (12%) in 2019, 12 (12%) in 2018, 9 (9%) in 2017, 8 (8%) in 2016, 2 (2%) in 2015, 4 (4%) in 2014, and 4 (4%) in 2013. The mean intervention duration was 111.4 (SD 132; range 7-730) days, with 27% (26/97) of studies lasting 1 to 4 weeks, 46% (46/97) of studies lasting between 1 and 3 months, 18% (17/97) of studies lasting between 3 and 12 months, and 9% (7/97) of studies lasting longer than a year. In 32% (31/97) of studies, monetary incentives were provided to the participants as compensation. The mean derived incentive value was US $105.42 (SD US $18.65; range US $7 to US $430).

### Characteristics of Study Populations

The total number of participants in the included studies evaluating specific mHealth apps was 72,046. The mean number of study participants was 750.5 (SD 2800.7; range 9-19,233). Of the 96 studies reporting exact participants numbers, 65 (68%) had <100 participants, 21 (22%) had 100 to 1000 participants, and only 10 (10%) studies had >1000 study participants. In several studies, most study participants had a pre-existing condition (82/97, 85%), with mental health conditions being the most prevalent (21/97, 22%), followed by obesity and being overweight (15/97, 15%), substance abuse (9/97, 9%), cancer (7/97, 7%), diabetes (5/97, 5%), CVD (5/97, 5%), and sleep disorder (4/97, 4%). In 15% (15/97) of studies, most participants were healthy. The overall mean age was 44.6 (SD 12.9; range 19.9-86) years, and the mean percentage of women was 62% (SD 22.8%; range 0%-100%).

### Characteristics of mHealth Apps

Of the 97 reviewed apps, 50 (51%) were available for both iOS and Android. The remaining apps were exclusively available on either iOS or Android platforms (both 17/97, 18%). The authors of 13% (13/98) of studies did not clearly outline on which platforms the apps were distributed. Of the 97 reviewed apps, 47 (48%) were publicly available, whereas 52 (54%) were exclusively available to study participants. Approximately 38% (37/97) of apps were developed by private commercial companies (eg, software companies), and 64% (62/97) of apps were developed by nonprofit organizations (eg, academic institutes). Of the studies that clearly outlined their study procedure, 34% (32/93) included personal contact with health personnel during the study as an intervention component. In comparison, 66% (61/93) of the apps provided only personal support in the app onboarding phase. Of the 54 studies evaluating the app’s effectiveness on a primary outcome, the authors of 34 (63%) studies highlighted their app as effective, and the authors of 20 (37%) studies highlighted their app as ineffective. The most common explanation of intended use, according to the authors, or derived from information on intervention design, was daily tracking (eg, daily diary entries; 36/97, 36%), followed by activity completion (eg, completion of a certain amount of coaching modules; 19/97, 20%), daily use (eg, daily log-in; 17/97, 18%), daily activity completion (6/97, 6%), weekly tracking (5/97, 5%), weekly use (4/97, 4%), activity completion+daily tracking (3/97, 3%), weekly use time (eg, using the app 1 hour per day; 2/97, 2%), prolonged use (eg, no inactivity for >2 weeks; 2/97, 2%), and biweekly tracking (1/97, 1%). Approximately 95% (92/96) of studies reported data on actual use based on objective app use data, and 4% (4/96) of studies reported data based on qualitative feedback from users. The mean adherence score across all interventions was 56.0% (SD 24.4%; range 2.6%-96.0%). The mean number of interactions within the study period amounted to 90.1 (SD 145.9; range 1-730) interactions. Of the 97 apps, 14 (14%) apps were intended for ≥2 daily interactions with the app, 53 (55%) apps were intended for 1 daily interaction, and 17 (18%) were intended for weekly interactions; in 13 (13%) apps, users only had to use the app once a month to be considered adherent.

### Characteristics of Health Domains

As displayed in Table 1, the included studies evaluating specific mHealth apps were categorized into the following health domains based on the individual app intervention focus: NCD self-management (17/97, 18%); mental health (20/97, 21%); substance use (9/97, 9%); nutrition (7/97, 7%); physical activity (6/97, 6%); weight loss (9/97, 9%); multicomponent lifestyle interventions (8/97, 8%); and mindfulness, including breathing and meditation interventions (9/97, 9%). Studies categorized in the domains of NCD self-management, mental health, and substance use were further subcategorized to report intervention and patient-related factors influencing intervention adherence at a more granular level. The studies (17/97, 18%) targeting NCD self-management were subcategorized into diabetes management (6/17, 35%), cancer management (5/17, 29%), respiratory disease management (3/17, 18%), CVD management (2/17, 12%), and medication adherence (1/17, 6%). Studies categorized in the mental health domain (20/97, 21%) were further divided into apps focusing on anxiety (2/20, 10%), depression (9/20, 45%), and multidisciplinary and other (9/20, 45%). The latter subdomain included other mental health problems such as bipolar disorders or combinations of various mental health problems. Studies in the substance use domain were further separated into apps addressing alcohol (2/9, 22%), tobacco (6/9, 67%), or a mix of various substances (1/9, 11%). Another 12% (12/97) of studies, which was a heterogeneous group targeting NCDs other than diabetes, cancer, CVD, respiratory disease, or medication adherence, were clustered into other NCDs (eg, intestinal and renal disease, insomnia, pain, venous leg ulcers, and dyslipidemia).

As outlined in Table 2, the mean number of participants was highest for studies that focused on substance use (2337.6, SD 6344.8; range 9-19,233) and lowest for other NCDs (54.8, SD 48.6; range 15-189), followed by weight loss interventions (73.2, SD 54.4; range 17-176).

As displayed in Table 3, the mean participant age was highest in apps targeting NCD self-management (57.7, SD 7.3; range 45-70.9 years) and lowest for mental health apps (35.9, SD 5.9; range 19.9-46.5 years).

Female populations were generally overrepresented (Table 4), especially in studies conducted on mindfulness interventions (76.7%, SD 20.2%; range 44.8%-100%). Only studies conducted on apps targeting substance use featured more men than women (percentage of women: mean 49.3%, SD 14.1%; range 27.7%-78%).

As outlined in Table 5, studies conducted on apps for weight loss had the most prolonged mean intervention duration (214, SD 216.3; range 65-730 days), and studies on nutrition had the shortest mean intervention duration (52.5, SD 55.7; range 7-172 days).

Table 6 shows distributions of total intended interactions with the apps over the course of the individual studies. The mean number of total intended interactions was highest for apps targeting weight loss (210.5, SD 213.3; range 52-730) and lowest for nutrition apps (31.8, SD 27.8; range 4-82.5).

The distribution of adherence scores by health domain is summarized in Table 7. The mean adherence scores were highest in the domain of other NCDs (69.9%, SD 18.5%; range 33.3%-90.5%), followed by multicomponent lifestyle interventions aimed at changing multiple behaviors simultaneously (61.3%, SD 22.5%; range 32.4%-96%). Apps from the substance use domain had the lowest adherence scores (46.1%, SD 33%; range 9.1%-84%).

Multimedia Appendix 4 [20,26-123] lists the identified intervention-related and patient-related factors with a positive or negative influence on adherence for each health domain in detail. The results per health domain are summarized in the following sections.

**Table 1:** Included studies evaluating specific mobile health apps categorized by health domain (N=97).

Health domains			Studies, n (%)		References
**NCD^a^ self-management**			17 (18)		[26-42]
	Diabetes management	6 (6)		[29-34]	
	Cancer management	5 (5)		[37-41]	
	Respiratory disease management	3 (3)		[26-28]	
	Cardiovascular disease management	2 (2)		[35,36]	
	Medication adherence	1 (1)		[42]	
**Mental health**			20 (21)		[43-62]
	Anxiety	2 (2)		[43,44]	
	Depression	9 (9)		[45-53]	
	Multidisciplinary and others	9 (9)		[54-62]	
**Substance use**			9 (9)		[63-71]
	Alcohol	2 (2)		[63,64]	
	Tobacco	6 (6)		[65-70]	
	Multidisciplinary and others	1 (1)		[71]	
Nutrition			7 (7)		[72-77,123]
Physical activity			6 (6)		[78-83]
Weight loss			9 (9)		[84-92]
Multicomponent lifestyle interventions			8 (8)		[93-100]
Mindfulness (including breathing and meditation)			9 (9)		[101-109]
Other NCDs			12 (12)		[110-121]
All domains			97 (100)		[26-121,123]

^a^NCD: noncommunicable disease.

**Table 2:** Number of participants by health domain in the included studies evaluating specific mobile health apps (N=97).

Health domains	Participants, N	Values, mean (SD)	Values, median (IQR)	Values, range
NCD^a^ self-management	15,111	888.9 (2433.3)	56 (113.5-31)	10-9051
Mental health	5710	285.5 (470.1)	81 (231.8-31)	14-1709
Substance use	21,038	2337.6 (6344.8)	99 (683.0-24)	9-19,233
Nutrition	13,042	2173.7 (5195.1)	22 (3342-12)	12-12,777
Physical activity	946	157.7 (147.5)	151 (301-22)	19-301
Weight loss	659	73.2 (54.4)	50 (120.5-28.5)	17-176
MLI^b^	2274	284.3 (531.9)	64.5 (331.3-29.3)	20-1561
Mindfulness	12,608	1400.9 (4031.5)	46 (128-21.5)	15-12,151
Other NCDs	658	54.8 (48.6)	44 (59.8-20.3)	15-189
All domains	72,046	750.5 (2800.7)	56 (129.5-26)	9-19,233

^a^NCD: noncommunicable disease.

^b^MLI: multicomponent lifestyle intervention.

**Table 3:** Age (years) of participants by health domain in the included studies evaluating specific mobile health apps (N=97).

Health domains	Values, mean (SD)	Values, median (IQR)	Values, range
NCD^a^ self-management	57.7 (7.3)	56.5 (64.2-52.6)	45.0-70.9
Mental health	35.9 (5.9)	36.6 (40.2-33.9)	19.9-46.5
Substance use	40.8 (9.6)	44.0 (48.8-35.3)	20.5-49.9
Nutrition	44.0 (16.6)	45.0 (60.0-27.2)	22.0-64.7
Physical activity	47.1 (15.2)	42.0 (63.6-38.0)	26.8-68.0
Weight loss	42.5 (11.0)	45.8 (49.6-35.2)	20.0-54.4
MLI^b^	43.7 (18.7)	39.0 (48.8-34.9)	23.6-86.0
Mindfulness	43.7 (14.7)	42.8 (52.8-33.5)	20.2-70.9
Other NCDs	45.6 (10.3)	43.6 (55.2-36.0)	34.0-64.9
All domains	44.6 (12.9)	42.9 (52.7-35.8)	19.9-86.0

^a^NCD: noncommunicable disease.

^b^MLI: multicomponent lifestyle intervention.

**Table 4:** Percentage of women by health domain in the included studies evaluating specific mobile health apps (N=97).

Health domains	Values (%), mean (SD)	Values (%), median (IQR)	Values (%), range
NCD^a^ self-management	53.6 (30.9)	50.3 (82.8-31.5)	0-100
Mental health	64.0 (16.5)	65.9 (72.6-58.4)	27.0-95.2
Substance use	49.3 (14.1)	50.5 (55.3-39.6)	27.7-78.0
Nutrition	67.7 (25.5)	71.5 (90.5-44.4)	31.0-94.0
Physical activity	60.6 (16.5)	64.0 (73.8-50.9)	30.4-73.9
Weight loss	60.7 (27.5)	68.9 (81.0-42.5)	1.3-85.0
MLI^b^	66.8 (12.5)	62.9 (78.6-60.0)	51.0-88.3
Mindfulness	76.7 (20.2)	80.2 (94.6-55.7)	44.8-100
Other NCDs	64.0 (26.4)	68.1 (87.5-45.8)	19.0-100
All domains	62.0 (22.8)	63.2 (78.9-48.0)	0-100

^a^NCD: noncommunicable disease.

^b^MLI: multicomponent lifestyle intervention.

**Table 5:** Intervention duration (days) by health domain in the included studies evaluating specific mobile health apps (N=97).

Health domains	Values, mean (SD)	Values, median (IQR)	Values, range
NCD^a^ self-management	129.1 (87.9)	91.3 (180-73.5)	30-365
Mental health	97.6 (137)	56 (78.8-30)	28-577.9
Substance use	109.2 (192.1)	56 (90.5-17.6)	14-615
Nutrition	52.5 (55.7)	28 (56-21)	7-172
Physical activity	74.3 (28)	81 (100-49)	28-100
Weight loss	214 (216.3)	180 (273.8-70)	56-730
MLI^b^	79.6 (50.7)	87 (91.3-33.3)	21-182.5
Mindfulness	107.1 (142.3)	42 (202.7-29)	21-365
Other NCDs	111 (126.6)	56 (159.4-31.5)	14-365
All domains	111.4 (132)	60.8 (100-30)	7-730

^a^NCD: noncommunicable disease.

^b^MLI: multicomponent lifestyle intervention.

**Table 6:** Number of intended app interactions by health domain in the included studies evaluating specific mobile health apps (N=97).

Health domains	Values, mean (SD)	Values, median (IQR)	Values, range
NCD^a^ self-management	141.6 (184.3)	90 (178.5-35)	6-615.4
Mental health	64.5 (124.6)	39.5 (57.5-6)	4-577.9
Substance use	51.3 (56.2)	19.1 (88.9-9)	4-170.7
Nutrition	31.8 (27.8)	21 (56-10)	4-82.5
Physical activity	60.8 (35.3)	67 (93.3-24)	12-100
Weight loss	210.5 (213.3)	168 (259.3-78)	52-730
MLI^b^	40.2 (36.3)	25.7 (81-6.8)	1-90
Mindfulness	144.1 (243.8)	30 (197.2-24.5)	21-730
Other NCDs	39.8 (35.3)	35 (65.5-5.8)	2-107.1
All domains	90.1 (145.9)	51 (90-20.1)	1-730

^a^NCD: noncommunicable disease.

^b^MLI: multicomponent lifestyle intervention.

**Table 7:** Adherence scores by health domain in the included studies evaluating specific mobile health apps (N=97).

Health domains	Values (%), mean (SD)	Values (%), median (IQR)	Values (%), range
NCD^a^ self-management	53.4 (24.7)	63.4 (72.5-25.5)	14.0-89.5
Mental health	56.6 (26.2)	61.0 (83.4-32.2)	15.0-91.5
Substance use	46.1 (33.0)	24.2 (81.1-18.0)	9.1-84.0
Nutrition	49.1 (32.1)	48.9 (84.3-26.8)	2.6-91.4
Physical activity	54.7 (16.6)	55.5 (71.3-39.9)	31.2-72.0
Weight loss	49.1 (21.5)	43.1 (61.6-38.2)	16.0-93.2
MLI^b^	61.3 (22.5)	56.1 (81.6-42.4)	32.4-96.0
Mindfulness	59.0 (18.5)	66.7 (73.7-37.2)	33.3-81.9
Other NCDs	69.9 (18.5)	72.4 (87.6-53.4)	33.3-90.5
All domains	56.0 (24.4)	60.4 (76.0-34.5)	2.6-96.0

^a^NCD: noncommunicable disease.

^b^MLI: multicomponent lifestyle intervention.

### NCD Self-management

#### Factors Influencing Adherence to NCD Self-management Apps

##### Diabetes Management

Intervention-related factors that positively influenced adherence to diabetes apps included automated and passive data collection within the app [31,34], customized reminders [31], game-based elements [32], and human-like app characteristics [32]. Manual data collection by users [31,34], lack of adjustment to users’ personal needs [34], and fast uptake of app activities after initiation [34] were associated with higher intervention dropouts and lower adherence.

Patient-related factors positively affecting adherence to diabetes apps were the following user characteristics: low extraversion [33], high educational level [33], openness to new experiences [33], exacerbated history of diabetes [33,34], and recent diagnosis of the disease [33,34]. Regarding the influence of user age on adherence, the results were contradictory. In one of the studies, users of older age were more adherent [34]; in contrast, another study found that older age was associated with weaker technology acceptance [33].

##### Cancer Management

Intervention-related factors that positively affected adherence to apps targeting patients with cancer included ongoing contact or telecoaching with health care professionals [37], personalization of users’ needs and cultural tailoring [38,39], and customizable reminders and notifications [39,40]. Furthermore, one of the studies showed that a continuous, nondelayed study course positively affected adherence [40].

The following user characteristics were positively associated with adherence: increased age, higher level of education, being married or in a relationship, and higher self-efficacy [37,40]. Furthermore, the active employment status of the users, leading to less available time for the intervention, was associated with lower adherence, especially among female users [38,40].

##### Respiratory Disease Management

In the context of intervention-related factors, personalization [27], app design, and ease of use [27,28], as well as personal contact or communication with a health care professional, were all positively associated with adherence [27,28].

Patient-related factors included the recruitment strategy and recruitment location. It was shown that users recruited personally and on site were more adherent than users recruited on the web or via social media [26]. Furthermore, the perceived health benefits and the sense of contributing to the science of the users were associated with better adherence [26]. The following user characteristics were negatively related to adherence: higher BMI [27], depression diagnosis [27], low educational level [27], and low smartphone literacy [28].

##### CVD Management

User interactions with the app through gamification, primarily through a personalized feedback and reward system, were associated with better adherence to apps targeting CVD self-management [36]. In addition, easy communication and data exchange between users and their health coaches positively affected adherence [35].

Patient-related factors that positively affected adherence were the user characteristics of hypertension diagnosis [122] and the high clinical demand of the patient [36]. Lack of technical experience with mobile devices and advanced age of patients were associated with lower adherence [35].

##### Medication Adherence

One of the studies using an app targeting medication adherence related to various NCDs did not include information about dedicated factors influencing adherence [42].

#### Quantitative Analysis of NCD Self-management Apps

Across the 5 considered subdomains, the mean adherence score was highest for one app targeting medication adherence (89.5%), followed by the subdomains cancer management (61.4%, SD 19.6%), respiratory disease management (52.7%, SD 21.5%), diabetes management (44.9%, SD 28.6%), and CVD management (41.5%, SD 28.9%). On average, adherence to mHealth apps targeting NCD self-management was 53.4% (SD 24.7%) across all the 5 health domains.

There was a strong positive, significant correlation between adherence score and the average age of study participants (*r*=0.624; 16/97, 16%; *P*=.01), which is consistent with the results of the 2 included publications [34,37]. There was no significant correlation between the level of personal support (*r_s_*_14_=0.150; *P*=.58) or gender (*r*=0.037; 16/97, 16%; *P*=.89) and adherence scores across studies targeting NCD self-management.

### Mental Health

#### Factors Influencing Adherence to Mental Health Apps

##### Anxiety

Compared with manual data collection by users, passive data collection was identified as an effective intervention-related factor in improving adherence in one of the studies [43]. It was also reported that technical problems negatively affected adherence and that iOS users had lower adherence than Android users [43].

##### Depression

The following intervention-related factors were positively linked with adherence to mHealth apps targeting depression: alternating intervention components and immediate feedback to maintain participant attention [49,52]; individualized features such as personalized representation of intervention progress, encouragement, and daily health tips [47]; offline app functionality and data plan independence [50]; a user-friendly and visually appealing app layout (eg, using a large font or highlighting essential app elements on the home screen) [53]; and evidence-based problem-solving therapies and content [47]. In contrast, a long study duration [49], competitive effects from other apps [50], and declining interest because of waiting times [47] were negatively linked to adherence.

The following user characteristics had a positive impact on adherence: local recruitment [45], ethnic minority background [48], and female gender [49]. In contrast, other characteristics negatively influenced adherence, including Latin America as a geographical origin [47], privacy concerns [47], low income [47], poor baseline depression [48,50] or anxiety, married relationship status [48], and lack of time [53]. In addition, remote recruitment (eg, via a web-based form [45,51]) was identified as a patient-related factor that negatively influences adherence.

##### Multidisciplinary and Others

The included studies identified individual functions that had a positive impact on adherence, such as crisis plans [58]; self-monitoring and visualization features [56]; tracking of stressful events [58]; tracking mood states with interactive mood charts [58]; visual feedback with personalized graphics interchange format images [62]; and dashboards with information on activity, sleep quality, mood development, and heart rate [61]. In addition, reminders through customizable push notifications were associated with better adherence [62]. Furthermore, the integration of health care professionals was positively linked to adherence [57]. Another study showed that integrating multiple intervention components and avoiding repetition and monotony had a positive impact on adherence [58]. Finally, lack of time for implementation and technical problems were negatively linked to adherence [58].

Patient-related factors positively influencing adherence included the following user characteristics: high IQ [56], increased age [58], increased risk of suicide [60], general interest in the app [54], and a trusting relationship between the person being treated and the organization providing the intervention [55]. In contrast, the following user characteristics negatively affected adherence: long treatment history [54], critical pre-existing conditions (eg, chronic psychotic illness [54]), increased overall mental health burden [56], increased mania-like symptoms [56], privacy concerns [57], or a perceived lack of usefulness of the app [58]. In addition, it was found that the app may lead to an unwanted reminder of one’s condition, which negatively affects adherence [58].

#### Quantitative Analysis of Mental Health Apps

The mean adherence score for the mental health apps was 56.6% (SD 26.2%). The mean adherence scores for apps offering support for anxiety, depression, and other mental health conditions were 67.4% (SD 33.8%), 45.3% (SD 28.1%), and 65.6% (SD 20.93%), respectively.

Regarding the positive effect of incorporating personal support from health care professionals into the intervention reported by Steare et al [57], the correlation between adherence score and the level of personal support for mental health apps, in general, was nonsignificant (*r_s_*_17_=0.230; *P*=.33). Compared with the qualitative data synthesis regarding the relationship between adherence score and average age [58], as well as the gender of student participants [49], no significant relationships were found quantitatively (*r*=0.096; 19/97, 20%; *P*=.70, and *r*=−0.149; 20/97, 21%; *P*=.53, respectively). Regarding the negative effect of long study durations [49], the correlation between adherence score and intervention duration in days was nonsignificant (*r*=−0.127; 20/97, 21%; *P*=.60). Compared with the difference between the iOS and Android operating systems mentioned in the qualitative analysis [43], the differences between the adherence score and smartphone operating system (*r_s_*_17_=0.450; *P*=.05) were likewise positive in the quantitative analysis.

### Substance Use

#### Factors Influencing Adherence to Substance Use Apps

##### Alcohol

Reminders in the form of daily push notifications were associated with better adherence [63]. Furthermore, personalization and customized content and features were positively linked to adherence [64]. A study also showed that gamification and gamified elements such as levels or rewards positively affected adherence [64]. Finally, variations and options in app design and offer within the app positively affected adherence [64].

The following user characteristics positively affected adherence as patient-related factors: female gender, low-risk alcohol consumption, high education level, reduced substance use, and increased age [64]. Doubts about efficacy and forgetfulness had a negative influence [64].

##### Tobacco

It was found that reminders in the form of daily push notifications positively affected adherence [69]. In addition, personalization and customized content in the app had the same impact [70]. The integration of and interaction with human coaches were positively associated with adherence [69]. Furthermore, the included studies found some specific features that increased adherence: tracking functions for self-monitoring (eg, as a diary [68,69]), a craving toolbox [69], all-general advice on quitting, and functions for stress and mood management [70].

Regarding patient-related factors, adherence was positively influenced by the following user characteristics: lower initial acceptance of cravings [67], younger age [66], and minimum level of digital skills among users [66].

##### Multidisciplinary and Others

One of the studies showed that the inclusion of several feedback modules is an effective technique for increasing adherence [71]. Otherwise, the included studies did not provide further information on factors influencing adherence [71].

#### Quantitative Analysis of Substance Use Apps

On average, the mHealth apps for substance use had an adherence score of 46.1% (SD 33.0%). Apps targeting alcohol use had a higher adherence score (51.5%, SD 38.6%) than those targeting tobacco use (38.0%, SD 32.7%). An app that combined both health behaviors had an adherence score of 83.4%.

Regarding the positive effect of incorporating human coaching into the intervention reported by Webb et al [69], the correlation between adherence score and the level of personal support for substance use apps, in general, was not significant (*r_s_*_6_=0.126; *P*=.77). Compared with the qualitative data synthesis in terms of the relationship between adherence score and average age [64,66] and gender of study participants [64], no significant relationships were found quantitatively (*r*=−0.094, 9/97, 9%, *P*=.81 and *r*=0.394, 9/97, 9%, *P*=.30, respectively).

### Nutrition

#### Factors Influencing Adherence to Nutrition Apps

Personalization of the intervention and certain app functions (personalized overview features of daily goals, recipe suggestions, lookup sections, camera or photograph-taking functions, and barcode scanners) were associated with better adherence to apps targeting nutrition [73,74,76]. Moreover, customized reminders and notifications and the integration of gamification elements combined with incentives enhanced engagement [73]. App handling and user-friendliness further positively influenced adherence [73,76]. The included studies pointed out the importance of the onboarding process, whereas enrollment methods with personal contact [123] had a positive impact, as well as appropriate guidance and tutorials at the beginning [73]. In addition, a relationship between uptake of the intervention activities and adherence was found, whereas starting the intervention on mornings and weekdays, in contrast to weekends, had a positive effect on the use of the mHealth app [75]. Finally, technical difficulties negatively affected adherence [72,73].

Several user characteristics had a positive influence. These were employment at a university [77], female gender [74], high degree of dietary preferences [75], and time and cognitive capacity devoted to the app [75]. The results were inconsistent concerning the age of the user. One of the studies associated older age with more adherence [74], whereas another showed the opposite [73].

#### Quantitative Analysis of Nutrition Apps

On average, the mHealth apps for nutrition had a mean adherence score of 49.1% (SD 32.1%). The positive effect of the inclusion of personal communication with health care professionals [73,75,123] was confirmed quantitatively, and the correlation between the adherence score and the level of personal support during the study period was strongly positive and significant (*r_s_*_4_=0.878; *P*=.02). Regarding the relationship between adherence score and age [73,74] and gender of study participants [74], no significant relationships were found quantitatively (*r*=−0.143; 7/97, 7%; *P*=.79, and *r*=0.234; 6/97, 6%; *P*=.66, respectively).

### Physical Activity

#### Factors Influencing Adherence to Physical Activity Apps

Of the 6 included studies targeting physical activity, 2 (33%) showed that customizable push notifications positively affected adherence [79,83]. In addition, the intervention-related factor, gamification, was associated with higher engagement [79]. Furthermore, social features, such as competitions, social comparison, and challenges, positively affected adherence [80,83]. In addition, personalization and customization were positively linked to adherence, especially customizable app functions regarding exercise plans, nutrition suggestions, and calorie lists [79,80,83]. Personal communication with and integration of health care professionals positively affected adherence [82]. Finally, technical difficulties negatively affected adherence [78].

The following user characteristics positively affected adherence: age [79], healthy BMI [79], and a positive attitude toward technology [78]. In contrast, users with increased disease severity, depressive symptoms, low quality of life, and poor access to transportation showed worse adherence [78]. In addition, privacy concerns and a lack of perceived benefits negatively influenced adherence [82].

#### Quantitative Analysis of Physical Activity Apps

On average, mHealth apps for improving physical activity had an adherence score of 54.7% (SD 16.6%). Regarding the positive effect of personal communication with health care professionals in the intervention [82], the correlation between the adherence score and level of personal support during the study period was not significant (*r_s_*_3_=0.289; *P*=.64). Compared with the qualitative data synthesis regarding the relationship between adherence score and average age [79], no significant correlations were found quantitatively (*r*=0.047; 6/97, 6%; *P*=.93).

### Weight Loss

#### Factors Influencing Adherence to Weight Loss Apps

The included studies focusing on weight loss apps identified a positive influence of reminders in the form of push notifications on adherence [88,92]. Just-in-time intervention components were associated with better adherence [87]. The same was found for newsfeeds with social components [88]. The studies highlighted that personal contact and integration of health care professionals positively influenced adherence [84,85,91]. Moreover, a correlation was found between high adherence and unlimited digital access via the app, as well as providing a data plan with no supplementary costs [89].

Several studies further identified the following user characteristics as patient-related factors to be positively linked to adherence: rural population [85], positive expectations regarding the intervention [88], prior experience with mHealth apps [88], a high sense of responsibility [86], and reinforcements through personal environment [85]. In contrast, dislike of the study equipment [86] and depression symptoms [89] adversely affected adherence.

#### Quantitative Analysis of Weight Loss Apps

On average, the mHealth apps for weight loss had an adherence score of 49.1% (SD 21.5%).

Regarding the positive effect of incorporating personal communication with health care professionals into the intervention [84,85,91], the quantitative analysis did not reveal a significant correlation (*r_s_*_7_=0.174; *P*=.65).

### Mindfulness

#### Factors Influencing Adherence to Mindfulness Apps

As most mindfulness apps that met the inclusion criteria of this review did not distinctively aim to treat a chronic mental condition but rather to increase well-being and reduce work-related stress, we categorized them as a separate category. Factors identified in the mindfulness domain may still be relevant for the mental health domain, as mindfulness-based therapy has been cited as a promising intervention for treating anxiety and depression in previous research [124].

The included studies reported that automated and interactive data collection and processing positively affected adherence [101,106]. The studies also showed that customizable features such as tracking stress and mood [109], visualizing personal progress, and immediate feedback positively affected adherence [103]. In addition, using in-app tutorials or video content was associated with better adherence [103]. Furthermore, time and place influenced adherence: users who used the app in the evening and at home were more adherent in the long term [106,107]. In contrast, extensive app interactions and lack of variety in the app content harmed adherence [107].

Regarding patient-related factors, the studies identified the following user characteristics to be positively related to adherence: increased age [102,108], positive expectations toward the app [106,108], intrinsic motivation [108], and a current physical diagnosis [106] in contrast to a mental health diagnosis.

#### Quantitative Analysis of Mindfulness Apps

On average, mHealth apps related to mindfulness had an adherence score of 59.0% (SD 18.5%). Regarding the positive relationship between adherence score and average age [102,108], the quantitative analysis also yielded a moderately positive but nonsignificant correlation (*r*=0.404; 9/97, 9%; *P*=.28).

### Multicomponent Lifestyle Interventions

#### Factors Influencing Adherence to Multicomponent Lifestyle Intervention Apps

Approximately 8% (8/97) of studies targeted mHealth apps focusing on multiple lifestyle behaviors (mostly a combination of physical activity, diet, weight loss, and sometimes sleep, stress, or headaches).

The included studies reported that the integration of health care professionals during the intervention, app usability, and language positively influenced adherence [95,98]. However, it was also shown that social networking and competition through social comparison in terms of physical activity level had a positive impact only if individuals had a healthy BMI [97]. In addition, app features such as audiovisual presentation of health-related information or reminders in the form of push notifications positively affected adherence [93,98]. Finally, personalization and tailoring of the app to customized needs (eg, through gamification) had a positive impact on adherence [97,98].

In addition to these factors, the following characteristics also positively affected adherence: increased age [95,100] and trust in the health care professionals of the intervention [95,100]. Finally, other characteristics negatively affected adherence. These included the lack of engagement of other participants in social comparison features [97], negative emotions related to self-monitoring during periods of weight [97], shift work schedules [98], and technical difficulties in using the app [93].

#### Quantitative Analysis of Multicomponent Lifestyle Intervention Apps

On average, mHealth apps targeting multicomponent lifestyle interventions had an adherence score of 61.3% (SD 22.5%).

The positive effect of integrating health care professionals as personal support into the intervention [95,98] could not be analyzed quantitatively as none of the included multicomponent lifestyle interventions offered consistent, continuous support by health care professionals (only during the onboarding phase). Regarding the mentioned positive relationship between adherence score and average age [95,100], the quantitative analysis also yielded a moderately positive but nonsignificant correlation (*r*=0.416; 8/97, 8%; *P*=.31).

### Other NCDs

#### Factors Influencing Adherence to Other NCD Apps

In a study on an mHealth app treating insomnia, better adherence was linked to ease of use and the easiness of therapy directives [110]. Another study on insomnia treatment found that easy access and reminder options or notifications had a positive impact [113]. In the field of chronic pain management and interventions, a study found a positive impact on adherence to microinteractions [111]. It was also shown in the same field that personalization had a positive impact [112]. In a study on the care of advanced chronic kidney disease, the integration of complementary visits to health care professionals or to a clinic showed a positive impact on adherence [117]. Furthermore, blood pressure and test result features and an automatic integrated transfer of blood pressure readings had a positive impact [117].

Regarding patient-related factors, a study on the treatment of irritable bowel syndrome showed that the simultaneous use of other technical devices positively affected adherence. Another study showed that high anxiety scores negatively affected adherence [119]. A study on the effects of a long-term smartphone-based self-monitoring intervention in patients with lipid metabolism disorders found that older age had a positive impact on adherence. In contrast, low acceptability, lack of time, health problems, and lack of motivation had a negative impact [118]. The user characteristic of the female gender positively influenced adherence in a study about an app for a lower leg physical activity intervention for individuals with chronic venous leg ulcers [114]. Furthermore, in a study on an mHealth app targeting inflammatory bowel disease, it was found that old age, low level of education, and lack of perceived usefulness negatively affected adherence [115].

#### Quantitative Analysis of Other NCD Apps

On average, mHealth apps categorized in the domain of *other NCDs* had an adherence score of 69.9% (SD 18.5%). Regarding the positive effect of complementary visits to health care professionals for the care of chronic kidney disease [117], the correlation between the adherence score and the level of personal support during the study period was nonsignificant (*r_s_*_10_=0.290; *P*=.36). The quantitative analysis supports the finding that female participants are more adherent [118] to some degree, as the correlation between the adherence score and mean percentage of female participants was moderately positive but nonsignificant (*r*=0.385; 12/97, 12%; *P*=.22). Regarding the relationship between adherence score and average age [118], the quantitative analysis yielded conflicting results (*r*=−0.619; 12/97, 12%; *P*=.03).

### Multi-Domain Review

One of the two included systematic reviews featured a multi-domain review focusing on uptake and engagement with mHealth apps in various health domains [20]. First, it showed a positive impact of goal setting, reward offerings, complementary web access, coping games, and self-monitoring. In addition, the low cost of the app helped increase acceptance [20].

The external influence of using an app through health care professionals, friends, and family or by reading user reviews was outlined as having a positive influence. Furthermore, community networking and the connection between the app and health professional support had a positive influence [20]. The study also found the following user characteristics to be positively linked to adherence: female gender, aged <44 years, living in urban areas, good educational level, high income, curiosity, higher health literacy, and app awareness [20]. In addition, interactivity, an established routine to use the app, and customization of the app had a positive impact [20]. In contrast, cognitive overload and unmet expectations negatively influenced adherence [20].

### Explorative Analysis of Adherence Scores

To gain further insights into the universal applicability of the results identified in individual health domains, a quantitative analysis was conducted on the total number of primary studies included. The analysis revealed a positive correlation between adherence score and level of personal support during the study period (*r_s_*_91_=0.199; *P*=.06). With respect to various user characteristics, the quantitative analysis did not find significant differences in either average age (*r*=0.105; 94/97, 97%; *P*=.32) or gender distribution (*r*=−0.031; 95/97, 97%; *P*=.77). Furthermore, no significant quantitative differences were found between healthy participants and participants with chronic diseases (*r_s_*_95_=−0.049; *P*=.63). A quantitative comparison of studies with monetary incentives and those without such incentives also revealed no significant effect on adherence scores (*r_s_*_92_=0.000; *P*=.99). However, the monetary value of the incentive, measured in US dollars, had a significant effect on the adherence score (*r*=0.465; 30/97, 31%; *P*=.01). Apps that were only offered in the context of scientific studies had a significantly higher adherence score than those that were publicly available via app stores (*r_s_*_95_=0.324; *P*=.001). The quantitative analysis did not find any significant differences between Android and iOS with regard to the adherence score (*r_s_*_83_=0.019; *P*=.87). Furthermore, the quantitative analysis showed a higher adherence score for apps developed by private app development companies than for those developed by public institutions or research groups (*r_s_*_95_=0.164; *P*=.11). The correlation between adherence score and intervention duration (*r*=−0.138; 97/97, 100%; *P*=.18) was negative but positive in relation to the number of intended app interactions per day (*r*=0.176; 97/97, 100%; *P*=.09). The comparison of adherence scores and the total number of intended app interactions (*r*=0.040, 97/97, 100%; *P*=.70) yielded no significant results. Studies with a higher number of app users had significantly lower adherence scores (*r*=−0.228; 96/97, 99%; *P*=.03).

## Discussion

### Intervention-Related Factors Influencing Adherence

Regarding the first research question, the intervention-related factors described in the following sections were identified most frequently across all health domains, suggesting universal applicability to increase mHealth app adherence relative to the intended use.

#### User-friendliness and Technical Stability

Approximately 18% (17/97) of studies from 6 health domains cited a user-friendly app design or technical stability as criteria for increased app use [27,28,43,50,53,58,72-74,76,78, 93-95,110,113,115,116]. The term *user-friendliness* describes a software interface that enables a simple, clean, intuitive, and reliable user experience (UX). App developers can thus promote adherence by making the app easy to use and providing a compelling and visually appealing app design (eg, by using sufficiently large fonts or highlighting essential app elements) [53]. Technical problems can be reduced through closed beta tests while optimizing the UX through user interface or UX design changes before app release. Accordingly, the quantitative analysis revealed higher adherence scores for apps created by private app development companies, which may have more technical expertise in developing and publishing apps than public institutions or research groups. Quantitative analysis also revealed higher adherence scores for apps developed by private companies, which may have more expertise across the value chain than public institutions or research groups. As most of the included mHealth apps had a pilot character (ie, developed by small academic teams with no or only short testing periods), it can be assumed that the current body of mHealth apps does not yet realize its full potential in terms of usability and technical stability, thus indicating the potential to improve adherence.

#### Personalization, Customization, and Tailoring

Approximately 16% (16/97) of studies from 8 different health domains reported a positive impact of personalized content on adherence [27,38,39,58,62,64,66,73,74,76,83,85,91,97,98,113]. This included individualized app features (such as a crisis plan), metrics, visualizations based on individual user data (eg, displaying intervention progress), personalized feedback and health suggestions, and individualized app content tailored to the needs and characteristics of users. These findings align with previous reviews that have summarized that an individualized app positively influences user engagement [20,125]. Accordingly, developers of mHealth apps should consider the target group’s characteristics and needs in the app design process and ideally make the app tailored to a specific user group, personalized to the individual, and customizable.

#### Individualized Reminders

In 13% (13/97) of studies across 8 different health domains, reminders, primarily realized through push notifications, were highlighted as an effective method of improving adherence to mHealth app interventions [31,39,40,62,63,69,73,79, 83,88,92-94,113]. Essential to the success of this technique is the consideration of users’ individual needs in terms of their schedule, as a user’s lack of time undermines adherence [38-40,53,58,118]. Ideally, users receive reminders when they are in a state of receptivity, which has also been suggested by previous research on just-in-time-adaptive interventions [126,127]. In this regard, working and leisure time schedules should be considered, and the timing of reminders should be adapted accordingly, particularly when patients are in the privacy and comfort of their homes. A recent review reported that reminders are helpful for people with busy schedules and when they are forgetful but also mentioned the risk that push notifications can threaten users’ social identity if they are received at an inappropriate time or place [20]. Therefore, users should be able to customize the reminders and adapt them to their circumstances.

#### Personal Support From Health Care Professionals

In 12% (12/97) of studies from 8 different health domains, personal support from a health care professional during the intervention was cited as a reason for improved adherence [28,35,37,57,69,82,84,91,95,98,117,123]. In this context, the integration of health care professionals past the initial app onboarding can be realized in various ways, including regular clinic visits, complementary telephone support, and communication options with health care professionals integrated into the app. In addition, apps can facilitate communication between patients and health care professionals through automated data exchanges. Quantitative analysis revealed a positive correlation between adherence and level of personal support during the study period for all health domains, which was also confirmed by previous studies [20,128]. Consequently, it can be assumed that hybrid systems that combine automated app content with elements of human support achieve higher adherence rates than those achieved by interventions without human support. Although the ideal ratio between human-computer interactions and sole human interactions in mHealth app interventions remains to be explored, new technologies such as conversational agents show promising results in simulating personal support without the need for human support and may enable increased levels of automation [129-132].

#### Elements of Gamification and Social Features

Elements of gamification were described as effective in increasing adherence in 12% (12/97) of studies across 6 different health domains [32,36,47,49,52,64,73,78-80,83,97,98]. These elements included levels, reward systems, social characters, contests, and leaderboards. This aligns with other reviews that list rewards and games as factors that positively affect adherence [20,125]. However, although game elements such as social competitions may increase engagement by encouraging others, the idea of defeating peers may also have a negative influence [20]. In 6% (6/97) of studies from 5 health domains, social features were found to positively affect adherence; for example, in the form of social networks, contests, leaderboards, or newsfeeds with social components [52,73,75,83,88,97]. However, social components should be included with caution and ideally tested, as social comparison with less-motivated participants can also harm adherence [97]. Accordingly, a recent review concluded that social contests increase engagement but may also have a negative effect [20]. In general, this study supports previous research outlining the positive effects of gamification and social features on DHIs [133-141].

#### Passive and Automated Data Collection, Processing, and Transmission

The positive influence of passive and automated data collection, processing, and transmission was reported in 5% (5/97) of studies from the health domains NCD self-management [31,34], other NCDs [117], mindfulness [101], and meditation [106]. Thus, developers can increase adherence by automating repetitive tasks to reduce user burden. In this regard, developers are advised to use smartphone sensor technology (eg, a camera to capture food data or accelerometer data to capture physical activity) and complementary devices (eg, a smartwatch to measure heart rate) to remove the repetition and monotony of intervention tasks, which are listed as reasons for nonadherence [58,60,70].

#### Monetary Incentives

Monetary incentives such as vouchers, lottery tickets, or direct cash contributions were given to participants in 31 of the 97 included primary studies [30,33,44,45,47,48,51,53,56,61, 62,64,65,68-72,77,81,88,90,98,101,104-106,108,109,114,116]. However, the qualitative synthesis did not yield results regarding the effect of such monetary incentives as an additional intervention component on adherence. The quantitative comparison between studies with monetary incentives and those without such incentives also found no effect on adherence. However, the monetary value of the incentive, measured in US dollars, had a significantly positive effect on adherence scores. In the context of these findings, app developers may consider whether monetary incentives are helpful and the level of compensation that is sufficient to achieve a relevant effect.

#### Other Intervention-Related Factors

Other notable intervention-related factors included integrating an app tutorial [73,103], presenting information in audiovisual formats [98], and offering a large variety of app content [64]. Approximately 3% (3/97) of studies also noted financial costs (eg, data plan use) as a barrier to adherence [20,89,92]. Therefore, developers may want to consider offering their mHealth app free of charge and only transferring large amounts of data when the device is connected to a wireless network. Other studies in this review also reported that time delays within the intervention, long intervention durations, low engagement of other participants, and competitive effects of other mHealth apps were associated with low adherence. The included studies also outlined data protection and user privacy as positively affecting adherence [20,47,57,82], which aligns with previous research calling developers to create robust and transparent mHealth apps that satisfy security and privacy demands to foster user acceptance and trust [142-145].

### Patient-Related Factors Influencing Adherence

Regarding the second research question, the patient-related factors described in the following section were identified.

#### Characteristics of Study Participants

Approximately 43% (42/97) of studies from 9 different health domains reported a wide variety of user characteristics that affect adherence, including age, gender, place of residence, marital status, health status, treatment history, education, employment status, income, and work hours [26-28,33-38,40,47-50,54,56,58,60,64,66,67,73-75,77-79, 85,86,88,89,95,98,100,102,106,108,114,115,118,119,122]. The quantitative analysis did not reveal significant effects of average age, gender distribution, or pre-existing conditions on adherence across all included apps. Consequently, the results from previous reviews, which indicate higher engagement among female or younger users, could not be replicated [20,125]. The specific health domains of NCD self-management and other NCDs showed a significant correlation between adherence scores and the average age of study participants, with the first one being positive and the latter being negative. Further research is required to understand the effects of sociodemographic characteristics and health status on adherence to mHealth apps. The findings of this study suggest that these effects may vary depending on the targeted health domain.

User characteristics associated with a low adherence were lack of technical competence, lack of health literacy, and lack of experience with mHealth apps, which could potentially be improved through preintervention training. Other negative factors, such as privacy concerns, low expectations of the app, and low trust in the health care professionals conducting the intervention, could potentially be challenged through personal communication in the onboarding phase (eg, by discussing the privacy policy or outlining intervention benefits). As lack of time on the users’ side was referenced to negatively affect adherence [53,58,107,118], helping patients with time management might also have a positive effect.

#### Type of Participant Recruitment

In 4% (4/97) of studies from the fields of NCD self-management [26], mental health [45,51], and nutrition [123], the user recruitment channel was mentioned as a relevant factor affecting adherence. Users who were made aware of the intervention on the web (eg, via social media) had lower adherence than users recruited locally and in person. This could explain why apps that were only offered in the context of studies on personal onboarding processes had significantly higher adherence scores than those publicly accessible via app stores. As the mHealth sector further matures and more mHealth apps are made available to the public via app stores, this factor might have an increasingly negative effect on overall adherence. Developers may overcome this issue by optimizing the UX of their mHealth app in the onboarding phase. As highlighted previously, offering personal support from health care professionals before and during the onboarding process is likely to increase adherence.

### Adherence Scores Across Health Domains

Regarding the third research question, this review outlined the differences between health domains in terms of adherence scores. The adherence score of all 97 included mHealth apps averaged 56.0% (SD 24.4%), representing a generally higher adherence level to mHealth apps than previous research suggests [9,10,146]. This could be attributed to the fact that some studies excluded participants who did not perform a certain level of activity (eg, downloading the app). Regarding the short intervention periods with a median of 60.8 days, it is questionable whether the included health apps could reach similar adherence scores in more prolonged studies. Adherence scores by health domain *were highest for the other NCDs (69.9%, SD 18.5%)*, a heterogeneous group of mHealth apps targeting less common NCDs such as intestinal and renal disease, insomnia, pain, venous leg ulcers, and dyslipidemia. Multicomponent lifestyle interventions aimed at changing multiple behaviors simultaneously (61.3%, SD 22.5%) had the second-highest adherence scores, whereas apps targeting substance use had the lowest (46.1%, SD 46.1%). The relatively low adherence levels of mHealth apps treating substance use could be explained by the nature of their intervention design, making it difficult to differentiate between nonadherent users and users who stopped using the app after a successful behavior change. Another explanation could be that substance use disorders are comorbid with depressive disorders. Several qualitative findings indicate that symptoms or a diagnosis of depression negatively affect adherence [27,48,50,78,89]. This also aligns with our findings that apps offering depression support had an even lower average adherence score of 45.3% (SD 28.1%).

Another explanation and potential bias for the difference in adherence scores are asymmetric distributions of trials compared with real-world applications within health domains, represented by the mean number of participants (substance use 2337.6, SD 6344.8; multicomponent lifestyle interventions 284.3, SD 531.9; and other NCDs 54.8, SD 48.6). In general, studies with a higher number of participants had significantly lower adherence scores (*r*=−0.228; 96/97, 99%; *P*=.03). Furthermore, apps that were only offered in the context of scientific studies had a significantly higher adherence score than those publicly available via app stores (*r_s_*_95_=0.324; *P*=.001). Surprisingly, these differences could not be explained by longer study duration (*r*=−0.138; 97/97, 100%; *P*=.18) or the number of intended app interactions (*r*=0.040; 97/97, 100%; *P*=.70). Thus, our study supports and provides quantitative evidence for previous findings outlining engagement within trials to significantly differ from real-world applications [147-149]. Further real-world longitudinal studies are necessary to explain these differences.

Although calculating an adherence score as the ratio between intended and actual use has several limitations, this exploratory approach enabled the quantification and comparison of adherence across different mHealth apps to a reasonable extent. The quantitative analysis of adherence scores yielded few significant results but fit qualitative findings regarding positive or negative influences on adherence in most instances, which supports the potential validity of the concept.

The results of explorative analysis based on adherence scores should be considered cautiously. Although we did not find a significant correlation between study duration or the number of intended app interactions and the adherence score, it is possible that other factors, such as patient characteristics, could influence adherence. Further research is needed to establish effective adherence measures. By reporting this novel quantified measure of adherence for individual studies and collectively for defined health domains, we hope researchers will test and challenge our approach, potentially developing more effective measures that help us quantify adherence and make it comparable across heterogeneous groups of mHealth apps.

### Limitations

This study had several limitations. The first limitation is the heterogeneity of the included studies. The studies differ in terms of the characteristics of the target populations (eg, type of pre-existing condition, age, gender, education level, comorbidities, employment status, and experience with mobile technologies) and study duration (a few weeks to over a year). In addition, mHealth apps within the studies and their intended use varied significantly. The problem of the undiversified reach of mHealth interventions, predominantly including female and White participants living in high-income countries [150], also accounts for this study. Approximately 90% (87/97) of these studies were conducted in North America, Australia, or Europe. Similarly, women were overrepresented, with an average proportion of 62% (SD 22.8%). Most included studies had a pilot character, with 80% (77/96) of the studies including <200 participants and 73% (71/97) of the studies having a duration of <100 days. Whether individual study results can be replicated in more controlled and longitudinal studies in the future is questionable. In addition, there were differences in terms of additional monetary incentives and the level of personal support complementary to the use of mHealth interventions. Moreover, the mHealth apps investigated in the studies showed substantial heterogeneity in their goals (eg, increasing physical activity and reducing tobacco consumption). Overall, this limited the generalizability of our results.

This limitation was overcome by categorizing the results into different health domains. However, mHealth apps also exhibited key differentiating characteristics within their health domain, such as their stage of development (prototype vs established app), developer (nonprofit vs private company), the use of peripheral devices (eg, a smartwatch for passive data collection), app features (eg, social features such as leaderboards and elements of gamification), or the level of quality (eg, in terms of the user interface, UX, or technical stability). Another limitation is that few studies considered individual intervention components separately and evaluated their effectiveness, which complicates the identification of intervention-related factors. A further limitation of this study is the inclusion of nonrandomized studies, which, on the one hand, allowed a more extensive consideration of objective app use data but, by contrast, precluded conducting a risk of bias assessment.

The calculation of adherence scores as the ratio between intended and actual use also implies some noteworthy drawbacks. The intended use of the mHealth app, which was derived from the study design or study author comments, was also heterogeneous and differed depending on the mHealth app design (eg, tracking daily symptoms or completing a certain number of coaching sessions), interaction frequency (eg, daily or weekly), and interaction duration (weeks to years). Furthermore, the intended use was rarely justified by applying theory, evidence, or rationale, which has also been addressed in previous studies [13]. In some instances, the intended use could be derived from the intervention design; however, many studies had to be excluded as the app’s intended use was unclear or not stated at all. However, it can be positively highlighted that the actual mHealth app use extracted for the adherence score was based on objective app use data in approximately 96% (92/96) of cases.

### Conclusions

This study contributes to the scarce scientific evidence on factors influencing adherence to mHealth apps and is the first to derive quantified adherence scores for various health domains to validate qualitative findings and explore adherence benchmarks.

This paper contains various detailed presentations of the central results. The most detailed presentation of adherence factors extracted from individual studies is outlined in Multimedia Appendix 4. We further classify the factors within the defined health domains and report the results collectively. Finally, we discuss the most common factors influencing adherence across all the health domains. As mHealth apps within health domains remain heterogeneous, we encourage readers to always consider information from the corresponding individual studies outlined in Multimedia Appendix 4 when implementing their interventions according to the factors reported in this review.

Our findings indicate that the following intervention-related factors positively influence mHealth app adherence: user-friendly and technically stable app design, customizable push notifications, personalized app content, passive data tracking, an integrated app tutorial, offering the app free of charge, and the integration of personal support into the mHealth app. Furthermore, gamification and social features show promising effects but may be limited to specific health domains. Time delays within the intervention, long intervention durations, low engagement of other participants, and the competitive effects of other mHealth apps were associated with low mHealth app adherence.

Regarding patient-related factors, the following user characteristics were associated with low mHealth app adherence: lack of technical competence, low health literacy, low self-efficacy, low education level, mental health burden, lack of experience with mHealth apps, privacy concerns, low expectations of the app, low trust in health care professionals conducting the intervention, and lack of time on the users’ side. Age, gender, and pre-existing condition were frequently mentioned factors but differed across and sometimes conflicted within the health domains. Furthermore, personal user recruitment appeared to positively influence adherence as opposed to web-based user recruitment.

The adherence score of the 97 included mHealth apps averaged 56.0% (SD 24.4%). Adherence scores were highest for mHealth apps targeting less common NCDs such as intestinal and renal disease, insomnia, pain, venous leg ulcers, and dyslipidemia. Multicomponent lifestyle interventions had the second-highest adherence scores, whereas apps targeting substance use had the lowest. Exploratory analysis of adherence scores revealed quantitative evidence for higher adherence rates within trials than in real-world applications.

Overall, research on the factors that positively or negatively influence adherence to mHealth apps is still limited. The underlying studies often had a pilot character with a short study duration, and the implementation of techniques was inconsistent. As most mHealth apps contain multiple intervention components, causal statements about individual factors are not possible and require more controlled and longitudinal studies in the future. To facilitate future research on mHealth app adherence; researchers should clearly outline and justify the app’s intended use, report objective data on actual use relative to the intended use; and ideally, provide long-term use and retention data. Further research is needed to establish effective adherence measures.

## Appendix

Multimedia Appendix 1 PRISMA (Preferred Reporting Items for Systematic Reviews and Meta-Analyses) checklist.

Multimedia Appendix 2 Applied search strategies.

Multimedia Appendix 3 Data extraction sheet.

Multimedia Appendix 4 Intervention-related and patient-related factors influencing adherence by health domain.

## Abbreviations

CVD: cardiovascular disease
DHI: digital health intervention
mHealth: mobile health
NCD: noncommunicable disease
PICOS: Population, Intervention, Comparison, Outcomes, and Study component
PRISMA: Preferred Reporting Items for Systematic Reviews and Meta-Analyses
UX: user experience
