# Supplementary material for: Factors Influencing Adherence to mHealth Apps for Prevention or Management of Noncommunicable Diseases: Systematic Review
Source: J Med Internet Res. 2022 May 25;24(5):e35371. doi: 10.2196/35371 (PMC9178451; doi:10.2196/35371)
Supplement: Multimedia Appendix 2 [file jmir_v24i5e35371_app2.docx]

## Multimedia Appendix 2: Applied Search Strategies

### Embase

#### Search strategy applied in Embase (January 3, 2021: 1334 results)

('mhealth':ti OR 'm-health':ti OR 'mobile health':ti OR 'mobile application*':ti OR 'mobile intervention*':ti OR 'mobile technolog*':ti OR app:ti OR apps:ti OR ios:ti OR iphone*:ti OR ipad*:ti OR android:ti OR smartphone*:ti OR 'smart phone*':ti OR 'cellular phone*':ti OR 'cellular telephone*':ti OR 'cell phone*':ti OR 'mobile phone*':ti OR 'mobile telephone*':ti OR 'car phone*':ti)

**OR**

('mobile phone'/exp OR 'smartphone'/exp)

**AND**

('engage*':ab,ti OR 'adhere*':ab,ti OR 'persist*':ab,ti OR 'retain*':ab,ti OR 'retention':ab,ti OR 'dropout*':ab,ti OR 'drop-out*':ab,ti OR 'disengage*':ab,ti OR 'dis-engage*':ab,ti OR 'usage':ab,ti OR 'nonusage':ab,ti OR 'non-usage':ab,ti OR 'intended use':ab,ti OR 'intended behavi*':ab,ti OR 'attrition':ab,ti OR 'compliance':ab,ti OR 'non-adhere*':ab,ti OR 'nonadhere*':ab,ti OR 'non-compli*':ab,ti OR 'noncompli*':ab,ti

OR 'cell phone use'/exp OR 'behavioral addiction'/exp OR 'patient attitude'/exp)

**NOT**

('infection'/exp)

**AND**

('acceptability stud*':ab,ti OR 'clinical trial*':ab,ti OR 'cohort stud*':ab,ti OR 'case-control stud*':ab,ti OR 'case control stud*':ab,ti OR 'controlled trial*':ab,ti OR 'cross over trial*':ab,ti OR 'cross-over trial*':ab,ti OR 'design evaluation’:ab,ti OR 'field stud*':ab,ti OR 'exploratory stud*':ab,ti OR 'exploratory investigation*':ab,ti OR 'empirical stud*':ab,ti OR 'empirical test*':ab,ti OR 'empirical examination*':ab,ti OR 'empirical analys*':ab,ti OR 'empirical assessment*':ab,ti OR 'empirical investigation*':ab,ti OR 'experiment*':ab,ti OR 'field stud*':ab,ti OR 'feasibility stud*':ab,ti OR 'feasibility test*':ab,ti OR 'longitudinal stud*':ab,ti OR 'longitudinal test*':ab,ti OR 'longitudinal examination*':ab,ti OR 'meta-analys*':ab,ti OR 'meta analys*':ab,ti OR 'micro-randomized trial*':ab,ti OR 'micro-randomised trial*':ab,ti OR 'mrt':ab,ti OR 'multilevel analys*':ab,ti OR ‘multi-level analys*’:ab,ti OR 'multimethod exploration*':ab,ti OR 'multi-method exploration*':ab,ti OR 'multimethod analys*':ab,ti OR 'multi-method analys*':ab,ti OR 'observational stud*':ab,ti OR 'proof of concept':ab,ti OR 'proof-of-concept':ab,ti OR 'pilot test*':ab,ti OR 'pilot stud*':ab,ti OR 'randomized trial*':ab,ti OR 'randomised trial*':ab,ti OR 'randomized stud*':ab,ti OR ‘randomised stud*’:ab,ti OR 'trial stud*':ab,ti OR 'systematic review*':ab,ti)

**AND**

([embase]/lim OR [medline]/lim OR [pubmed-not-medline]/lim) AND [2007-2021]/py AND [humans]/lim AND ([article]/lim OR [article in press]/lim) AND [english]/lim AND ([adult]/lim OR [young adult]/lim OR [middle aged]/lim OR [aged]/lim OR [very elderly]/lim)

#### Embase Output

('mhealth':ti OR 'm-health':ti OR 'mobile health':ti OR 'mobile application*':ti OR 'mobile intervention*':ti OR 'mobile technolog*':ti OR app:ti OR apps:ti OR ios:ti OR iphone*:ti OR ipad*:ti OR android:ti OR smartphone*:ti OR 'smart phone*':ti OR 'cellular phone*':ti OR 'cellular telephone*':ti OR 'cell phone*':ti OR 'mobile phone*':ti OR 'mobile telephone*':ti OR 'car phone*':ti OR 'mobile phone'/exp OR 'smartphone'/exp) AND ('engage*':ab,ti OR 'adhere*':ab,ti OR 'persist*':ab,ti OR 'retain*':ab,ti OR 'retention':ab,ti OR 'dropout*':ab,ti OR 'drop-out*':ab,ti OR 'disengage*':ab,ti OR 'dis-engage*':ab,ti OR 'usage':ab,ti OR 'nonusage':ab,ti OR 'non-usage':ab,ti OR 'intended use':ab,ti OR 'intended behavi*':ab,ti OR 'attrition':ab,ti OR 'compliance':ab,ti OR 'non-adhere*':ab,ti OR 'nonadhere*':ab,ti OR 'non-compli*':ab,ti OR 'noncompli*':ab,ti OR 'cell phone use'/exp OR 'behavioral addiction'/exp OR 'patient attitude'/exp) NOT 'infection'/exp AND ('acceptability stud*':ab,ti OR 'clinical trial*':ab,ti OR 'cohort stud*':ab,ti OR 'case-control stud*':ab,ti OR 'case control stud*':ab,ti OR 'controlled trial*':ab,ti OR 'cross over trial*':ab,ti OR 'cross-over trial*':ab,ti OR 'design evaluation':ab,ti OR 'exploratory stud*':ab,ti OR 'exploratory investigation*':ab,ti OR 'empirical stud*':ab,ti OR 'empirical test*':ab,ti OR 'empirical examination*':ab,ti OR 'empirical analys*':ab,ti OR 'empirical assessment*':ab,ti OR 'empirical investigation*':ab,ti OR 'experiment*':ab,ti OR 'field stud*':ab,ti OR 'feasibility stud*':ab,ti OR 'feasibility test*':ab,ti OR 'longitudinal stud*':ab,ti OR 'longitudinal test*':ab,ti OR 'longitudinal examination*':ab,ti OR 'meta-analys*':ab,ti OR 'meta analys*':ab,ti OR 'micro-randomized trial*':ab,ti OR 'micro-randomised trial*':ab,ti OR 'mrt':ab,ti OR 'multilevel analys*':ab,ti OR 'multi-level analys*':ab,ti OR 'multimethod exploration*':ab,ti OR 'multi-method exploration*':ab,ti OR 'multimethod analys*':ab,ti OR 'multi-method analys*':ab,ti OR 'observational stud*':ab,ti OR 'proof of concept':ab,ti OR 'proof-of-concept':ab,ti OR 'pilot test*':ab,ti OR 'pilot stud*':ab,ti OR 'randomized trial*':ab,ti OR 'randomised trial*':ab,ti OR 'randomized stud*':ab,ti OR 'randomised stud*':ab,ti OR 'trial stud*':ab,ti OR 'systematic review*':ab,ti) AND ([embase]/lim OR [medline]/lim OR [pubmed-not-medline]/lim) AND [2007-2021]/py AND [humans]/lim AND ([article]/lim OR [article in press]/lim) AND [english]/lim AND ([adult]/lim OR [young adult]/lim OR [middle aged]/lim OR [aged]/lim OR [very elderly]/lim)

### WebOfScience

#### Search strategy applied in WebOfScience (January 3, 2021: 763 results)

(TI=(“mhealth” OR “m-health” OR “mobile health” OR “mobile application*” OR “mobile intervention*” OR “mobile technolog*” OR “app” OR “apps” OR “ios” OR “iphone*” OR “ipad*” OR “android” OR “smartphone*” OR “smart phone*” OR “cellular phone*” OR “cellular telephone*” OR “cell phone*” OR “mobile phone*” OR “mobile telephone*” OR “car phone*”)

**AND**

(TS=(“engage*” OR “adhere*” OR “persist*” OR “retain*” OR “retention” OR “dropout*” OR “drop-out*” OR “disengage*” OR “dis-engage*” OR “usage” OR “nonusage” OR “non-usage” OR “intended use” OR “intended behavi*” OR “attrition” OR “compliance” OR “non-adhere*” OR “nonadhere*” OR “non-compli*” OR ”noncompli*”) )

**AND**

(TS=(“acceptability stud*“ OR “clinical trial*“ OR “cohort stud*“ OR “case-control stud*“ OR “case control stud*“ OR “controlled trial*“ OR “cross over trial*“ OR “cross-over trial*“ OR “design evaluation“ OR “field stud*“ OR “exploratory stud*“ OR “exploratory investigation*“ OR “empirical stud*“ OR “empirical test*“ OR “empirical examination*“ OR “empirical analys*“ OR “empirical assessment*“ OR “empirical investigation*“ OR “experiment*“ OR “field stud*“ OR “feasibility stud*“ OR “feasibility test*“ OR “longitudinal stud*“ OR “longitudinal test*“ OR “longitudinal examination*“ OR “meta-analys*“ OR “meta analys*“ OR “micro-randomized trial*“ OR “micro-randomised trial*“ OR “mrt“ OR “multilevel analys*“ OR “multi-level analys*“ OR “multimethod exploration*“ OR “multi-method exploration*“ OR “multimethod analys*“ OR “multi-method analys*“ OR “observational stud*“ OR “proof of concept“ OR “proof-of-concept“ OR “pilot test*“ OR “pilot stud*“ OR “randomized trial*“ OR “randomised trial*“ OR “randomized stud*“ OR “randomised stud*“ OR “trial stud*“ OR “systematic review*“) )

**NOT**

(SU=(“Anesthesiology” OR “Anatomy & Morphology” OR “Anesthesiology” OR “Biochemistry & Molecular Biology” OR “Biodiversity & Conservation” OR “Biotechnology & Applied Microbiology” OR “BiophysicsCell Biology” OR “Developmental Biology” OR “Entomology” OR “Environmental Sciences & Ecology” OR “Evolutionary Biology” OR “Fisheries” OR “Genetics & Heredity” OR “Infectious Diseases” OR “Integrative & Complementary Medicine” OR “Legal Medicine” OR “Marine & Freshwater Biology” OR “Medical Ethics” OR “Medical Informatics” OR “Microbiology” OR “Mycology” OR “Neurosciences & Neurology” OR “Paleontology” OR “Parasitology” OR “Pediatrics” OR “Plant Sciences” OR “Toxicology” OR “Transplantation” OR “Tropical Medicine” OR “Veterinary Sciences” OR “Virology” OR “Zoology” OR “Arts & Humanities” OR “Physical Sciences”) )

#### WebOfScience Output

((TI=(“mhealth” OR “m-health” OR “mobile health” OR “mobile application*” OR “mobile intervention*” OR “mobile technolog*” OR “app” OR “apps” OR “ios” OR “iphone*” OR “ipad*” OR “android” OR “smartphone*” OR “smart phone*” OR “cellular phone*” OR “cellular telephone*” OR “cell phone*” OR “mobile phone*” OR “mobile telephone*” OR “car phone*”) ) AND (TS=(“engage*” OR “adhere*” OR “persist*” OR “retain*” OR “retention” OR “dropout*” OR “drop-out*” OR “disengage*” OR “dis-engage*” OR “usage” OR “nonusage” OR “non-usage” OR “intended use” OR “intended behavi*” OR “attrition” OR “compliance” OR “non-adhere*” OR “nonadhere*” OR “non-compli*” OR ”noncompli*”) ) AND (TS=(“acceptability stud*“ OR “clinical trial*“ OR “cohort stud*“ OR “case-control stud*“ OR “case control stud*“ OR “controlled trial*“ OR “cross over trial*“ OR “cross-over trial*“ OR “design evaluation“ OR “field stud*“ OR “exploratory stud*“ OR “exploratory investigation*“ OR “empirical stud*“ OR “empirical test*“ OR “empirical examination*“ OR “empirical analys*“ OR “empirical assessment*“ OR “empirical investigation*“ OR “experiment*“ OR “field stud*“ OR “feasibility stud*“ OR “feasibility test*“ OR “longitudinal stud*“ OR “longitudinal test*“ OR “longitudinal examination*“ OR “meta-analys*“ OR “meta analys*“ OR “micro-randomized trial*“ OR “micro-randomised trial*“ OR “mrt“ OR “multilevel analys*“ OR “multi-level analys*“ OR “multimethod exploration*“ OR “multi-method exploration*“ OR “multimethod analys*“ OR “multi-method analys*“ OR “observational stud*“ OR “proof of concept“ OR “proof-of-concept“ OR “pilot test*“ OR “pilot stud*“ OR “randomized trial*“ OR “randomised trial*“ OR “randomized stud*“ OR “randomised stud*“ OR “trial stud*“ OR “systematic review*“) ) NOT (SU=(“Anesthesiology” OR “Anatomy & Morphology” OR “Anesthesiology” OR “Biochemistry & Molecular Biology” OR “Biodiversity & Conservation” OR “Biotechnology & Applied Microbiology” OR “BiophysicsCell Biology” OR “Developmental Biology” OR “Entomology” OR “Environmental Sciences & Ecology” OR “Evolutionary Biology” OR “Fisheries” OR “Genetics & Heredity” OR “Infectious Diseases” OR “Integrative & Complementary Medicine” OR “Legal Medicine” OR “Marine & Freshwater Biology” OR “Medical Ethics” OR “Medical Informatics” OR “Microbiology” OR “Mycology” OR “Neurosciences & Neurology” OR “Paleontology” OR “Parasitology” OR “Pediatrics” OR “Plant Sciences” OR “Toxicology” OR “Transplantation” OR “Tropical Medicine” OR “Veterinary Sciences” OR “Virology” OR “Zoology” OR “Arts & Humanities” OR “Physical Sciences”) )) AND LANGUAGE: (English) AND DOCUMENT TYPES: (Article)

Refined by: Open Access: ( OPEN ACCESS )

Timespan: 2007-2020. Indexes: SCI-EXPANDED, SSCI, A&HCI, CPCI-S, CPCI-SSH, BKCI-S, BKCI-SSH, ESCI, CCR-EXPANDED, IC.

### Scopus

#### Search strategy applied in SCOPUS (January 3, 2021: 598 results)

( ( TITLE ( “mhealth” OR “m-health” OR “mobile health” OR “mobile application*” OR “mobile intervention*” OR “mobile technolog*” OR “app” OR “apps” OR “ios” OR “iphone*” OR “ipad*” OR “android” OR “smartphone*” OR “smart phone*” OR “cellular phone*” OR “cellular telephone*” OR “cell phone*” OR “mobile phone*” OR “mobile telephone*” OR “car phone*”)) )  AND  ( TITLE-ABS ( “engage*” OR “adhere*” OR “persist*” OR “retain*” OR “retention” OR “dropout*” OR “drop-out*” OR “disengage*” OR “dis-engage*” OR “usage” OR “nonusage” OR “non-usage” OR “intended use” OR “intended behavi*” OR “attrition” OR “compliance” OR “non-adhere*” OR “nonadhere*” OR “non-compli*” OR ”noncompli*” ) )  AND  ( TITLE-ABS ( “acceptability stud*“ OR “clinical trial*“ OR “cohort stud*“ OR “case-control stud*“ OR “case control stud*“ OR “controlled trial*“ OR “cross over trial*“ OR “cross-over trial*“ OR “design evaluation“ OR “field stud*“ OR “exploratory stud*“ OR “exploratory investigation*“ OR “empirical stud*“ OR “empirical test*“ OR “empirical examination*“ OR “empirical analys*“ OR “empirical assessment*“ OR “empirical investigation*“ OR “experiment*“ OR “field stud*“ OR “feasibility stud*“ OR “feasibility test*“ OR “longitudinal stud*“ OR “longitudinal test*“ OR “longitudinal examination*“ OR “meta-analys*“ OR “meta analys*“ OR “micro-randomized trial*“ OR “micro-randomised trial*“ OR “mrt“ OR “multilevel analys*“ OR “multi-level analys*“ OR “multimethod exploration*“ OR “multi-method exploration*“ OR “multimethod analys*“ OR “multi-method analys*“ OR “observational stud*“ OR “proof of concept“ OR “proof-of-concept“ OR “pilot test*“ OR “pilot stud*“ OR “randomized trial*“ OR “randomised trial*“ OR “randomized stud*“ OR “randomised stud*“ OR “trial stud*“ OR “systematic review*“) )  AND  ( LIMIT-TO ( PUBYEAR ,  2021 )  OR  LIMIT-TO ( PUBYEAR ,  2020 )  OR  LIMIT-TO ( PUBYEAR ,  2019 )  OR  LIMIT-TO ( PUBYEAR ,  2018 )  OR  LIMIT-TO ( PUBYEAR ,  2017 )  OR  LIMIT-TO ( PUBYEAR ,  2016 )  OR  LIMIT-TO ( PUBYEAR ,  2015 )  OR  LIMIT-TO ( PUBYEAR ,  2014 )  OR  LIMIT-TO ( PUBYEAR ,  2013 )  OR  LIMIT-TO ( PUBYEAR ,  2012 )  OR  LIMIT-TO ( PUBYEAR ,  2011 )  OR  LIMIT-TO ( PUBYEAR ,  2010 )  OR  LIMIT-TO ( PUBYEAR ,  2009 )  OR  LIMIT-TO ( PUBYEAR ,  2008 )  OR  LIMIT-TO ( PUBYEAR ,  2007 ) )  AND  ( LIMIT-TO ( LANGUAGE ,  "English" ) )  AND  ( LIMIT-TO ( EXACTKEYWORD ,  "Adult" ) )

#### Scopus Output

( ( TITLE ( "mhealth" OR "m-health" OR "mobile health" OR "mobile application*" OR "mobile intervention*" OR "mobile technolog*" OR "app" OR "apps" OR "ios" OR "iphone*" OR "ipad*" OR "android" OR "smartphone*" OR "smart phone*" OR "cellular phone*" OR "cellular telephone*" OR "cell phone*" OR "mobile phone*" OR "mobile telephone*" OR "car phone*" ) ) ) AND ( TITLE-ABS ( "engage*" OR "adhere*" OR "persist*" OR "retain*" OR "retention" OR "dropout*" OR "drop-out*" OR "disengage*" OR "dis-engage*" OR "usage" OR "nonusage" OR "non-usage" OR "intended use" OR "intended behavi*" OR "attrition" OR "compliance" OR "non-adhere*" OR "nonadhere*" OR "non-compli*" OR "noncompli*" ) ) AND ( TITLE-ABS ( "acceptability stud*" OR "clinical trial*" OR "cohort stud*" OR "case-control stud*" OR "case control stud*" OR "controlled trial*" OR "cross over trial*" OR "cross-over trial*" OR "design evaluation" OR "field stud*" OR "exploratory stud*" OR "exploratory investigation*" OR "empirical stud*" OR "empirical test*" OR "empirical examination*" OR "empirical analys*" OR "empirical assessment*" OR "empirical investigation*" OR "experiment*" OR "field stud*" OR "feasibility stud*" OR "feasibility test*" OR "longitudinal stud*" OR "longitudinal test*" OR "longitudinal examination*" OR "meta-analys*" OR "meta analys*" OR "micro-randomized trial*" OR "micro-randomised trial*" OR "mrt" OR "multilevel analys*" OR "multi-level analys*" OR "multimethod exploration*" OR "multi-method exploration*" OR "multimethod analys*" OR "multi-method analys*" OR "observational stud*" OR "proof of concept" OR "proof-of-concept" OR "pilot test*" OR "pilot stud*" OR "randomized trial*" OR "randomised trial*" OR "randomized stud*" OR "randomised stud*" OR "trial stud*" OR "systematic review*" ) ) AND ( LIMIT-TO ( PUBYEAR , 2021 ) OR LIMIT-TO ( PUBYEAR , 2020 ) OR LIMIT-TO ( PUBYEAR , 2019 ) OR LIMIT-TO ( PUBYEAR , 2018 ) OR LIMIT-TO ( PUBYEAR , 2017 ) OR LIMIT-TO ( PUBYEAR , 2016 ) OR LIMIT-TO ( PUBYEAR , 2015 ) OR LIMIT-TO ( PUBYEAR , 2014 ) OR LIMIT-TO ( PUBYEAR , 2013 ) OR LIMIT-TO ( PUBYEAR , 2012 ) OR LIMIT-TO ( PUBYEAR , 2011 ) OR LIMIT-TO ( PUBYEAR , 2010 ) OR LIMIT-TO ( PUBYEAR , 2009 ) OR LIMIT-TO ( PUBYEAR , 2008 ) OR LIMIT-TO ( PUBYEAR , 2007 ) ) AND ( LIMIT-TO ( LANGUAGE , "English" ) ) AND ( LIMIT-TO ( EXACTKEYWORD , "Adult" ) )

### ACM

#### Search strategy applied in ACM (January 3, 2021: 129 results)

"mhealth" OR "m-health" OR "mobile health" OR "mobile application*" OR "mobile intervention*" OR "mobile technolog*" OR "app" OR "apps" OR "ios" OR "iphone*" OR "ipad*" OR "android" OR "smartphone*" OR "smart phone*" OR "cellular phone*" OR "cellular telephone*" OR "cell phone*" OR "mobile phone*" OR "mobile telephone*" OR "car phone*"

**AND**

"engage*" OR "adhere*" OR "persist*" OR "retain*" OR "retention" OR "dropout*" OR "drop-out*" OR "disengage*" OR "dis-engage*" OR "usage" OR "nonusage" OR "non-usage" OR "intended use" OR "intended behavi*" OR "attrition" OR "compliance" OR "non-adhere*" OR "nonadhere*" OR "non-compli*" OR "noncompli*"

**AND**

"acceptability stud*" OR "clinical trial*" OR "cohort stud*" OR "case-control stud*" OR "case control stud*" OR "controlled trial*" OR "cross over trial*" OR "cross-over trial*" OR "design evaluation" OR "field stud*" OR "exploratory stud*" OR "exploratory investigation*" OR "empirical stud*" OR "empirical test*" OR "empirical examination*" OR "empirical analys*" OR "empirical assessment*" OR "empirical investigation*" OR "experiment*" OR "field stud*" OR "feasibility stud*" OR "feasibility test*" OR "longitudinal stud*" OR "longitudinal test*" OR "longitudinal examination*" OR "meta-analys*" OR "meta analys*" OR "micro-randomized trial*" OR "micro-randomised trial*" OR "mrt" OR "multilevel analys*" OR "multi-level analys*" OR "multimethod exploration*" OR "multi-method exploration*" OR "multimethod analys*" OR "multi-method analys*" OR "observational stud*" OR "proof of concept" OR "proof-of-concept" OR "pilot test*" OR "pilot stud*" OR "randomized trial*" OR "randomised trial*" OR "randomized stud*" OR "randomised stud*" OR "trial stud*" OR "systematic review*"

#### ACM Output

*[[Abstract: "mhealth"] OR [Abstract: "m-health"] OR [Abstract: "mobile health"] OR [Abstract: "mobile application*"] OR [Abstract: "mobile intervention*"] OR [Abstract: "mobile technolog*"] OR [Abstract: "app"] OR [Abstract: "apps"] OR [Abstract: "ios"] OR [Abstract: "iphone*"] OR [Abstract: "ipad*"] OR [Abstract: "android"] OR [Abstract: "smartphone*"] OR [Abstract: "smart phone*"] OR [Abstract: "cellular phone*"] OR [Abstract: "cellular telephone*"] OR [Abstract: "cell phone*"] OR [Abstract: "mobile phone*"] OR [Abstract: "mobile telephone*"] OR [Abstract: "car phone*"]] AND [[Abstract: "engage*"] OR [Abstract: "adhere*"] OR [Abstract: "persist*"] OR [Abstract: "retain*"] OR [Abstract: "retention"] OR [Abstract: "dropout*"] OR [Abstract: "drop-out*"] OR [Abstract: "disengage*"] OR [Abstract: "dis-engage*"] OR [Abstract: "usage"] OR [Abstract: "nonusage"] OR [Abstract: "non-usage"] OR [Abstract: "intended use"] OR [Abstract: "intended behavi*"] OR [Abstract: "attrition"] OR [Abstract: "compliance"] OR [Abstract: "non-adhere*"] OR [Abstract: "nonadhere*"] OR [Abstract: "non-compli*"] OR [Abstract: "noncompli*"]] AND [[Abstract: "acceptability stud*"] OR [Abstract: "clinical trial*"] OR [Abstract: "cohort stud*"] OR [Abstract: "case-control stud*"] OR [Abstract: "case control stud*"] OR [Abstract: "controlled trial*"] OR [Abstract: "cross over trial*"] OR [Abstract: "cross-over trial*"] OR [Abstract: "design evaluation"] OR [Abstract: "field stud*"] OR [Abstract: "exploratory stud*"] OR [Abstract: "exploratory investigation*"] OR [Abstract: "empirical stud*"] OR [Abstract: "empirical test*"] OR [Abstract: "empirical examination*"] OR [Abstract: "empirical analys*"] OR [Abstract: "empirical assessment*"] OR [Abstract: "empirical investigation*"] OR [Abstract: "experiment*"] OR [Abstract: "field stud*"] OR [Abstract: "feasibility stud*"] OR [Abstract: "feasibility test*"] OR [Abstract: "longitudinal stud*"] OR [Abstract: "longitudinal test*"] OR [Abstract: "longitudinal examination*"] OR [Abstract: "meta-analys*"] OR [Abstract: "meta analys*"] OR [Abstract: "micro-randomized trial*"] OR [Abstract: "micro-randomised trial*"] OR [Abstract: "mrt"] OR [Abstract: "multilevel analys*"] OR [Abstract: "multi-level analys*"] OR [Abstract: "multimethod exploration*"] OR [Abstract: "multi-method exploration*"] OR [Abstract: "multimethod analys*"] OR [Abstract: "multi-method analys*"] OR [Abstract: "observational stud*"] OR [Abstract: "proof of concept"] OR [Abstract: "proof-of-concept"] OR [Abstract: "pilot test*"] OR [Abstract: "pilot stud*"] OR [Abstract: "randomized trial*"] OR [Abstract: "randomised trial*"] OR [Abstract: "randomized stud*"] OR [Abstract: "randomised stud*"] OR [Abstract: "trial stud*"] OR [Abstract: "systematic review*"]] AND [Publication Date: (01/01/2007 TO 12/31/2020)]*
