# Supplementary material for: Factors Influencing Adherence to mHealth Apps for Prevention or Management of Noncommunicable Diseases: Systematic Review
Source: J Med Internet Res. 2022 May 25;24(5):e35371. doi: 10.2196/35371 (PMC9178451; doi:10.2196/35371)
Supplement: Multimedia Appendix 4 [file jmir_v24i5e35371_app4.docx]

## Multimedia Appendix 4: Intervention-Related and Patient-Related Factors Influencing Adherence by Health Domain

### NCD-Self-Management

|  | **NCD-Self-Management*** | | | |
| --- | --- | --- | --- | --- |
|  | **Respiratory**  **Disease** | **Diabetes** | **Cardiovascular**  **Disease** | **Cancer** |
| References | 1 (Hui et al. 2019)  2 ([Rodriguez Hermosa et al.](#_ENREF_1) 2020)  3 (Deng et al. 2020) | 4 (Sittig et al. 2020)  5 (Dugas et al. 2018)  6 (Padhye and Wang. 2015)  7 (Gong et al. 2020)  8 (Su et al. 2020)  9 (Böhm et al. 2020) | 10 (Choi et al. 2020)**  11 (Harzand et al. 2018)  12 (Kaplan et al. 2017) | 13 (Crafoord et al. 2020)  14 (Baik et al. 2020)  15 (Benze et al. 2019)  16 (Min et al. 2014)  17 (Greer et al. 2020) |
| Intervention-Related Factors | **Positive influence**   - Personalization^2^ - Tracking features such as exercise tracking, monitoring of weight, food intake^2^ - Timely feedback from health care providers^3^ - Easy access and handling^2,3^ | **Positive influence**   - Automated and passive data collection^6,9^ - Customized, automatic reminders/notifications^6^ - Game-based elements^7^ - Human-like app characteristics^7^   **Negative influence**   - Suboptimal matching to the needs of the users (users experience that the app does not exactly fit their needs)^9^ - Many/high demands and tasks in the app^9^ - Quick uptake of activities in the app after initiation^9^ | **Positive influence**   - Easy data sharing / communication between user & coach^11^ - Dashboard (allowing remote monitoring of the patient)^11^ - Frequent and in-depth app interactions of users through gamification (such as personalized feedback and reward system)^12^ | **Positive influence**   - Persuasive design^13^ - Frequent, continuous exchange or telecoaching with healthcare professionals^13^ - Cultural tailoring^14^ and adaptations to the needs of patients^15^ (eg, individualization of the content). - Notifications or reminders at configurable and personalized times^15,16^ - Absence of study lag time^16^ |
| Patient-Related Factors | **Positive influence**   - Personal invitation and recommendations from known and trusted people^1^ - Perceived health benefits (eg, by improving control over disease, providing practical help managing symptoms, and increasing awareness of the disease)^1^ - Sense of contributing to science^1^   **Negative influence**   - Recruitment via social media^1^ - Higher BMI^2^ - Depression Diagnosis^2^ - Low education level^2^ - Low smartphone literacy^3^ | **Positive influence**   - Diabetes and recent diabetes diagnosis^8, 9^ - Young age^8^ - Increased age (older people may be motivated to apply their newly acquired skills)^9^ - Low extraversion or increased introversion (more likely to use this app instead of a social network)^8^ - High education level^8^ - Openness to new experiences^8^ - User self-efficacy (patients' belief in their own ability to manage their complex chronic health condition in the context of app use)^4^ | **Positive influence**   - Patients with hypertension^10^ - High clinical need^12^   **Negative influence**   - Lack of technical experience with smartphones^11^ - Increased age^11^ | **Positive influence**   - Advanced age^13^ - High education level^13^ - Being married or living with a partner^13^ - High self-efficacy^16^   **Negative influence**   - Memory impairment^13^ - Increased feeling of illness^13^ - High baseline comorbitities^13^ - Active employment status (less private time)^14^ - Female gender and currently employed^16^ |

* One study classified as Medication Adherence did not include information about dedicated factors influencing adherence and is thus not listed here.

** This systematic review was included for the synthesis of factors influencing adherence, but not for the quantitative analysis as it did not evaluate a specific mHealth app.

### Mental Health

|  | **Mental Health** | | |
| --- | --- | --- | --- |
|  | Anxiety | Depression | Multidisciplinary |
| References | 1 (Pham et al. 2016)  2 (Miller et al. 2020) | 3 (Dahne et al. 2019)  4 (Mantani et al. 2017)  5 (Pratap et al. 2018)  6 (Arean et al. 2016)  7 (Economides et al. 2020)  8 (Hung et al. 2016)  9 (Deady et al. 2020)  10 (Furukawa et al. 2018)  11 (Collins et al. 2020) | 12 (Bonet et al. 2020)  13 (Tighe et al. 2017)  14 (Ryan et al. 2020)  15 (Steare et al. 2020)  16 (Hidalgo-Mazzei et al. 2018)  17 (Proudfoot et al. 2013)  18 (Porras-Segovia et al. 2020)  19 (Ponzo et al. 2020)  20 (Beard et al. 2020) |
| Intervention-Related Factors | **Positive influence**   - Remote study measure obtainment^1^ | **Positive influence**   - Apps based on evidence-based problem-solving therapy^5^ - Daily health tips for overcoming depressed mood^5^ - Reward structure (eg, through personalized illustrations of study progress, personal encouragement)^5^ - A wide variety of intervention components^7,10^ (to help maintain participants’ attention and reduce fatigue) - In-session feedback with gamification-elements^7^ - Offline app functionality / data plan independency^8^ - %-Calculator (diff. Socratic questions, and more specific demand of self-reflection)^10^ - “Nice!” Voting button (analog to Facebook’s ‘like’-button, creating an atmosphere of a therapeutic community) ^10^ - User-friendly and visually engaging app design (eg, accessibility through large fonts or improved navigation through refined home screen with highlighting of important app elements and challenge flow)^11^   **Negative influence**   - Time delays causing waiting time^5^ - Long study duration (fatigue, boredom)^7^ - Lack of immediate feedback^8^ - Competition effect of other applications^8^ | **Positive influence**   - Self-monitoring and visualization features^14,16,19^ (eg, symptom tracking and subsequent inclusion in medical decision making^14^; ability to add personally stressful events^16^; new methods for tracking mood state with interactive mood charts^16^; dashboards with info on activity, sleep quality, mood trend, heart rate, etc.^19^). - Support through healthcare professionals^15^ - Personalization^20^ (eg, personalized crisis plan^16^) - Variety of psychoeducational messages^16^ - Personalized notifications/ reminders^20^ - Feedback with graphics interchange format (GIF)^20^ (Postexercise feedback was presented alongside an encouraging GIF image (eg, famous actor giving a thumbs-up) representing people with diverse ages and ethnoracial backgrounds.)   **Negative influence**   - Lack of integration of healthcare professionals (eg, no access)^15^ - Repetition and monotony of questions/tasks^16, 18^ - Lack of time for testing^16^ - Technical problems^16^ |
| Patient-Related Factors | **Negative influence**   - Technical issues^1^ - iOS Platform^1^ | **Positive influence**   - Local recruitment^3^ - Racial/ethnic minorities background^6^ - Female gender^7^   **Negative influence**   - Remote recruitement^3^, online-assessment^8^, and an online onboarding process^9^ - Hispanic/latino origin^5^ - Privacy concerns^5^ - Low income^5^ - High baseline depression or anxiety^6, 8^ - High baseline anxiety^6^ - Being married^6^ - Long study duration (fatigue, boredom)^7^ - Lack of time^11^ | **Positive influence**   - Inherent interest in the app^12^ - Acceptance and cultural appropriateness^13^ (willingness of participants to recommend the app to others in their networks) - Trusting relationships between users and provider ^13^ - Increased IQ^14^ - Increased age^16^ - Suicidal patients^18^   **Negative influence**   - Patients with long history of treatment^12^ - Illness condition (eg, chronic psychotic illness^12^, increased overall mental health burden^14^, increased mania-like symptoms^14^) - Data privacy concerns^15^ - Perceived lack of utility^16^ - App being an undesired reminder of one's condition^16^ |

### Substance Use

|  | **Substance Use** | | |
| --- | --- | --- | --- |
|  | Alcohol | Tobacco | Multidisciplinary |
| References | 1 (Bell et al. 2020)  2 (Laurens et al. 2020) | 3 (Herbec et al. 2019)  4 (Pallejà-Millán et al. 2020)  5 (Zeng et al. 2016)  6 (Vilardaga et al. 2019)  7 (Webb et al. 2020)  8 (Hébert et al. 2020) | 9 (Witkiewitz et al. 2014) |
| Intervention-Related Factors | **Positive influence**   - Daily push notifications^1^ - Personalized and customized content^2^ - Gamification (eg, levels and rewards)^2^ - Variation in app-appearance^2^ | **Positive influence**   - Personalized and customized content^4^ - Daily push notifications^7^ - Combination of in-app and human coaching^7^ - Keeping a diary, self-monitoring as well as a craving toolbox ^5,6,7^ - Education and CBT content^7,8^ - General advice (for quitting)^8^ - Features for stress and mood management^8^   **Negative influence**   - Monotony of the app as well as repeated surveys^8^ | **Positive influence**   - Greater “dose” of modules in the intervention^9^ |
| Patient-related Factors | **Positive influence**   - Female gender^2^ - Not-increased alcohol consumption^2^ - Good education^2^ - Decreased substance use^2^ - Older age^2^   **Negative influence**   - Doubts of effectiveness^2^ - Simple forgetfulness of the users^2^ | **Positive influence**   - Young age^4^ - Minimum of digital skills^4^ - Lower initial acceptance for cravings^5^ | / |

### Nutrition

|  | **Nutrition** |
| --- | --- |
| References | 1 (Chang et al. 2020)  2 (Chen et al. 2017)  3 (Hendrie et al. 2020)  4 (Helander et al. 2014)  5 (Linardon et al. 2020)  6 (Orlemann et al. 2018)  7 (Robinson et al. 2013) |
| Intervention-Related Factors | **Positive influence**   - Positive peer-feedback, high level of feedback on initiation^4^ - Enrolment methods with personal contact^5^ - Easy operation, app-handling^6^ - Matching, user-friendly and personalizable app functions (like overview feature of daily goal, recipe suggestions, lookup sections, camera or photo-taking function, Barcode scanning)^2, 3, 6^ - Guidance and in-app tutorial^2^ - Customized reminders^2^ - Entertaining functions (particularly Incentives and rewards)^2^ - Social networking motivation factors^2^   **Negative influence**   - Technical problems^1,2^ - First time app-usage on weekends (poorer diet quality, higher kcal intake leads to less incentive to track with the app)^4^ |
| Patient-related Factors | **Positive influence**   - Working at a university (higher appreciation of research)^7^ - High degree of dietary preferences^4^ - Female gender^3^ - Old age^3^ - Young age^2^ - Individualized pace approach thanks to the app^5^ - First time app-usage on weekdays or in the morning/ day-time^4^ - Time/cognitive capacity devoted on initiation^4^   **Negative influence**   - First time app-usage on weekends (poorer diet quality, higher kcal intake leads to less incentive to track with the app)^4^ - Achievement of defined goals (users are less dependent on the app once their goal has been achieved)^3^ - Usage behavior becomes routine (making other mechanics necessary)^3^ - Online recruitment of users^5^ |

### Physical Exercise

|  | **Physical Exercise** |
| --- | --- |
| References | 1 (Bentley et al. 2020)  2 (Edney et al. 2020)  3 (Edney et al. 2019)  4 (Kramer et al. 2020)  5 (Landers et al. 2020)  6 (Luhanga et al. 2018) |
| Intervention-Related Factors | **Positive influence**   - Behavior-self-monitoring (step calendar use)^3^ - Targeted and tailored informationen^6^ - Gamified features (for example social features^2,3^, competitions and challenges^3,6^ or leaderboards^2^) - App features (such as exercise plans, nutrition suggestions, food calories list^6^) - Customizable and tailored push notifications/ prompts^3,6^ - Behavior change techniques (BCTs)^5^ - Social comparison and inter-group competition^6^   **Negative influence**   - Lack of direct contact/interaction^5^ - Technical problem^1^ |
| Patient-related Factors | **Positive influence**   - Old age^3^ - Healthy BMI^3^ - Interest in technology^1^   **Negative influence**   - Attitude toward technology (use is perceived as daunting/overwhelming)^1^ - Perceived lack of benefit^1^ - Increased disease severity^1^ - Low quality of life^1^ - Symptoms of depression^1^ - Poor access to transportation (reason for not participating and not completing the study).^1^ - Privacy concerns^5^ - Interruption of the normal routine^1^ |

### Weight Loss

|  | **Weight Loss** |
| --- | --- |
| References | 1 (Allen et al. 2013)  2 (Bennett et al. 2018)  3 (Carter et al. 2013)  4 (Forman et al. 2019)  5 (Hales et al. 2016)  6 (Ifejika et al. 2020)  7 (Stephens et al. 2017)  8 (Svetkey et al. 2015)  9 (Tanaka et al. 2018) |
| Intervention-Related Factors | **Positive influence**   - Increased accountability^7^ - Digital access 24/7 via app and providing data plan with no suppl. costs^6,9^ - Personal contact and coaching beyond self-monitoring alone^1,8^ - Data integration with dieticians and GPs^2^ - Personalized Feedback^2^ - Friendly and regular reminders/ prompts^5,9^ - Just-in-time interventions (more suitable to less flexible dietary interventions)^4^ - Newsfeed with social component^5^ - Behavior-Change-Technique elements^8^ - Customization^8^ - Short daily intervention rhythm^4^ |
| Patient-related Factors | **Positive influence**   - Rural population (increased phone dependency, as the primary and sole internet connection)^2^ - Positive expectations^5^ - Prior experience with mHealth apps^5^ - Sense of responsibility, feeling of obligation^3^ - Amplified accountability and reinforcements through personal environment^2^   **Negative influence**   - Dislike of study equipment^3^ - Depression Diagnosis^6^ |

### Multicomponent Lifestyle Interventions

|  | **Multicomponent Lifestyle Interventions** |
| --- | --- |
| References | 1 (Horsch et al. 2017)  2 (Göransson et al. 2020)  3 (Murawski et al. 2019)  4 (Laranjo et al. 2020)  5 (Oftedal et al. 2019)  6 (Recio-Rodriguez et al. 2016)  7 (Seng et al. 2018) |
| Intervention-Related Factors | **Positive influence**   - Reminders and push-notifications^1^ - Easy usability (especially for older people)^2^ - Relevant health information^2^ - Competition through social comparison (with more active participants in terms of physical activity)^4^ - Personalization, tailoring to individual needs and gamification^4, 5^ - Simple language^5^ - Information in audio-visual formats^5^ - Motivation through involvement of healthcare professionals in the intervention^2, 5^   **Negative influence**   - Upward social comparison in individuals with higher BMI^4^ |
| Patient-Related Factors | **Positive influence**   - Trust in healthcare professionals^2^ - Old age^2,7^ - Goal agreement^4^ - Generally increased investment in a treatment^7^   **Negative influence**   - Lack of motivation or engagement from other individuals^4^ - Negative emotions associated with self-monitoring during periods of weight gain^4^ - Technical difficulties when using the app^1^ - Shift working hours^5^ |

### Mindfulness

|  | **Mindfulness (including Meditation and Breathing)** |
| --- | --- |
| References | 1 (Avalos et al. 2020)  2 (Bostock et al. 2019)  3 (Chandler et al. 2020)  4 (Champion et al. 2018)  5 (Goldberg et al. 2020)  6 (Huberty et al. 2019)  7 (Lengacher et al. 2018)  8 (Mahlo and Windsor. 2020)  9 (Walsh et al. 2019) |
| Intervention-Related Factors | **Positive influence**   - Automatic, interactive progress-tracking^1,6^ - In-app audio and video guide tutorials^3^ - Immediate feedback^3^ - Summarizing graphs or charts (allows comparison over weeks/months)^3^ - Use of the application in the evening (fewer barriers, does not require additional time or motivation)^6^ - Possibility to carry out the intervention at home in privacy^7^ - Monitoring of mood and stress (increased self-awareness and acceptance of negative emotions)^9^   **Negative influence**   - Too much requested practice time^7^ - Little/no new content and ideas^7^ |
| Patient-related Factors | **Positive influence**   - Old age^2,8^ - Physical health diagnosis complementary to physical health diagnosis^6^ - Positive expectation towards the app^6^ and the feel of being interesting, enjoyable, valuable, and useful^8^ - Intrinsic motivation^8^ |

### Other Non-communicable Diseases

|  | Other Non-communicable Diseases |
| --- | --- |
| References | 1 (Aji et al. 2020)  2 (Gentili et al. 2020)  3 (Hauser-Ulrich et al. 2020)  4 (Kang et al. 2017)  5 (Kelechi et al. 2020)  6 (McCombie et al. 2020)  7 (Minen et al. 2020)  8 (Ong et al. 2016)  9 (Steinert et al. 2020)  10 (Weerts et al. 2020)  11 (Isetta et al. 2017)  12 (Zia et al. 2016) |
| Intervention-Related Factors | **Positive influence**   - Microinteractions^2^ - Personalization^3^ - Easy access^4^ - Reduced chance of losing data^4^ (eg, paper diaries are lost more frequently) - Regular clinic visits complementary to the digital intervention^8^ - Ease of use, straightforward therapy directives^1^ - Reminder option as well as notifiactions^4^ - Blood pressure (BP) and test results feature^8^ - Automatic transfer of BP readings^8^   **Negative influence**   - Static flow of interaction^3^ - Increased practice demand (homework)^7^ - Technical problems^6,7^ - Rigorous alpha testing (Enhanced user friendliness and comprehensibility)^2^ |
| Patient-related Factors | **Positive influence**   - Simultaneous use of other technical devices by user^10^ - Older age^9^ - Female gender^5^   **Negative influence**   - High anxiety score^10^ - Old age^6^ - Low level of education^6^ - Lack of acceptability or perceived usefulness^6,9^ - Lack of time^9^ - Health problems^9^ - Lack of motivation^9^ |

### Multi-Domain Reviews

|  | Multi-Domain Reviews |
| --- | --- |
| References | (Szinay et al. 2020) |
| Intervention-Related Factors | **Positive influence**   - Offering rewards - Goal setting - Completement with web access (Higher engagement rate with app + website compared to app-only or web-only.) - Low cost of an app (Factor affecting acceptance) - Coping games (Useful as distraction activities to cope with craving (smoking) or distress)). - Self-monitoring   **Inconclusive results**   - Statistical information, not conclusive: Useful as a behavioral reinforcement but not useful during ‘bad days’ with progress viewing evoking negative emotions / discouragement. - Reminders, not conclusive: Useful for people with busy schedules and for forgetfulness. Tailored health message = small increase in engagement. Not useful for behaviors with stigma (eg, alcohol), reminders would threaten the users’ social identity when received at an inappropriate time/place. - User guidance (eg, instructions), not conclusive: Can increase engagement, but off-putting and unnecessary for long-term engagement by producing negative emotions (eg, annoyance) once the knowledge regarding app functionality has been gathered. - Embedded social media, not conclusive: depending on the individual’s attitude / target behavior - Social competition, not conclusive: Increases engagement by receiving encouragement from others but an idea of defeating each other might prompt discouragement - Available health information, not conclusive: increased engagement. HI that focuses on negative aspects of past behavior that cannot be modified might trigger negative emotions (eg, regrets). - Popularity indicators (eg, ranking on app stores), not conclusive: may not correspond with effectiveness, and rapid disengagement is common). |
| Patient-related Factors | **Positive influence**   - Social influence (Positive influence to use an app through healthcare professionals, friends and family, or by reading user reviews) - Connections between app & health practitioner support - Community networking - Female gender - Younger than 44 years - Living in an urban area - Good education level - High income - App awareness - Established routine - Interactivity and positivity of tone - A personalized app - Personal experience related to NCDs - Curiosity - Health literacy   **Negative influence**   - Cognitive overload - Unmet expectations |
